# Workplace bullying, speak-up climate and perceived patient safety compromise among radiographers practising in the United Kingdom

**DOI:** 10.3389/frhs.2026.1924715

**Published:** 2026-10-19

**Authors:** Chidi Ollawa, Olanrewaju Lawal, Naman Julka-Anderson, Rajuno Eteng, Basil Nnaemeka Ezenwuba, Robert Meertens, Edozie Iweka

**Affiliations:** 1 Department of Radiology, North Cumbria Integrated Care NHS Foundation Trust, Carlisle, United Kingdom; 2 School of Health and Rehabilitation Sciences, Health Sciences University, Bournemouth, United Kingdom; 3 Department of Radiology, Mid and South Essex NHS Foundation Trust, Basildon, United Kingdom; 4 Department of Radiology, University Hospitals of Leicester NHS Trust, Leicester, United Kingdom; 5 Department of Medical Imaging, Sheffield Teaching Hospitals NHS Foundation Trust, Sheffield, United Kingdom; 6 Department of Health and Care Professions, University of Exeter Faculty of Health and Life Sciences, Exeter, United Kingdom; 7 University of Bath, Bath, England, United Kingdom

**Keywords:** organisational voice, patient safety climate, radiographer wellbeing, speak-up culture, workplace bullying and harassment, workplace culture

## Abstract

**Introduction:**

Workplace bullying and harassment (WBH) may undermine speak-up climate, suppress organisational voice, and negatively affect patient safety. However, quantitative evidence examining these relationships within radiography practice remains limited. This study aimed to examine associations among WBH exposure, speak-up climate, and perceived compromise of patient safety among UK radiographers.

**Methods:**

A UK-wide cross-sectional survey of 385 HCPC-registered radiographers, recruited via non-probability sampling, was conducted using an online questionnaire that incorporated the Negative Acts Questionnaire–Revised (NAQ-R), patient safety impact items, and speak-up climate indicators. Composite Safety Impact and Speak-Up Climate scores were constructed. Internal consistency was assessed using Cronbach's alpha. Associations were evaluated using Spearman correlations with Benjamini-Hochberg false discovery rate correction. Multivariable linear regression models with HC3-robust standard errors were used to assess adjusted associations. Exploratory mediation analyses using 5,000 bootstrap samples examined the indirect association involving Speak-Up Climate. Optional free-text responses were analysed using inductive qualitative content analysis.

**Results:**

Greater WBH exposure was associated with poorer Speak-Up Climate (rho = 0.51, q < 0.001) and greater perceived compromise in patient safety (rho = 0.17, q = 0.003). In covariate-adjusted models, the NAQ-R mean score was associated with poorer Speak-Up Climate (*N* = 369; B = 0.688, 95% CI 0.550–0.825, *p* < 0.001; R^2^ = 0.250, adjusted R^2^ = 0.231) and greater perceived Safety Impact (*N* = 358; B = 0.316, 95% CI 0.179–0.453, *p* < 0.001; R^2^ = 0.118, adjusted R^2^ = 0.096). When Speak-Up Climate was added to the Safety Impact model, the adjusted NAQ-R association was attenuated and no longer statistically significant (*N* = 357; B = 0.052, 95% CI −0.095–0.199, *p* = 0.488). By contrast, poorer Speak-Up Climate remained associated with greater perceived Safety Impact (B = 0.378, 95% CI 0.255–0.501, *p* < 0.001; model R^2^ = 0.230, adjusted R^2^ = 0.208). An exploratory 5,000-resample bootstrap analysis identified an indirect association via Speak-Up Climate (B = 0.264, 95% bootstrap CI 0.169–0.373). Complete-case sensitivity analyses produced materially unchanged estimates. Qualitative findings included the following categories: organisational silence and psychological unsafety, leadership and organisational accountability and building psychologically safe workplace cultures

**Conclusion:**

Speak-Up Climate statistically accounted for part of the observed cross-sectional association between WBH exposure and perceived compromise of patient safety. Longitudinal research is required to evaluate temporal or causal relationships.

**Implications for practice:**

Psychologically safe reporting environments and anti-retaliation cultures are plausible organisational priorities that warrant prospective evaluation.

## Introduction

Patient safety fundamentally depends on healthcare professionals’ ability to raise concerns, report errors, and challenge unsafe practices. Psychological safety, defined as the shared belief that individuals can speak up without fear of negative consequences, is a critical determinant of effective safety culture (1). When psychological safety is compromised, healthcare workers may withhold concerns, increasing the risk of error concealment, impaired organisational learning, and patient harm (2).

Workplace bullying and harassment (WBH) represent organisational risk factors that may undermine psychological safety. WBH involves repeated exposure to negative acts such as humiliation, intimidation, or social exclusion in situations characterised by power imbalance (3). Extensive research across healthcare professions has demonstrated associations between workplace bullying and reduced psychological well-being, increased burnout, and impaired organisational functioning (4, 5).

Importantly, emerging evidence suggests that WBH is associated with safety-related behaviours, including incident reporting, willingness to raise concerns, and engagement with organisational safety processes. Studies in nursing and medicine have shown that exposure to WBH is associated with reduced incident reporting, fear of retaliation, and disengagement from safety behaviours (6, 7). Psychological distress and impaired workplace climate have also been associated with poorer patient safety outcomes (8). These findings suggest that WBH may represent not only a workforce wellbeing issue but also a potential threat to patient safety.

Despite these concerns, radiography remains under-researched in relation to patient safety and workplace culture. Radiographers work at critical diagnostic points in patient care, where errors in imaging acquisition, protocol adherence, or communication can have downstream consequences for diagnosis and treatment. Radiography practice often involves high workloads, shift work, and professional hierarchies that may affect psychological safety and willingness to raise concerns (9, 10). However, quantitative evidence on the relationship among workplace bullying, speak-up climate, and perceived compromise of patient safety in radiography remains very limited.

This evidence gap is particularly important given ongoing workforce pressures. UK imaging services face substantial workforce shortages and increasing diagnostic demand (10). National workforce surveys continue to report high levels of workplace bullying and low confidence in organisational response to concerns (11). Organisational failures in the NHS have repeatedly highlighted cultures of fear and silence as contributing factors to patient harm (12). Understanding organisational and psychosocial determinants of safety culture is therefore essential for improving patient safety.

Reframing workplace bullying as an organisational safety risk, rather than solely an individual wellbeing issue, has important implications. Drawing on psychological safety theory (1), organisational silence frameworks (2), and psychosocial safety climate theory (13), the present study conceptualises workplace bullying as an organisational stressor that may limit safety-related speak-up behaviours and contribute to perceived compromise in patient safety.

This study aims to examine associations among workplace bullying and harassment, Speak-Up Climate, and perceived patient safety compromise among UK radiographers. Therefore, the study sought to address the following research questions: *1. Is exposure to workplace bullying associated with a poorer speak-up climate? 2. Is exposure to workplace bullying linked to a higher perception of patient safety compromise? 3. To what extent is the association between workplace bullying and perceived patient safety compromise statistically accounted for by Speak-Up Climate?*

## Methods

### Study design

This study forms Phase 3 of the *Towards Inclusive Radiography* research programme and uses the same cross-sectional cohort as the companion Phase 2 manuscript. The companion paper investigates the psychological and occupational consequences of workplace bullying, including psychological distress, presenteeism, sickness absence and retention-related outcomes, whereas the present manuscript focuses on organisational safety by examining the Speak-Up Climate Score, Safety Impact Score, reporting behaviour, organisational prevention strategies and an exploratory indirect statistical association involving Speak-Up Climate. Although participant characteristics and selected prevalence estimates are necessarily shared across manuscripts describing the same cohort, the research questions, outcome variables, regression models, qualitative analyses, figures and principal findings reported here are distinct. Supplementary Table S1 provides a detailed comparison.

### Sample size

The Health and Care Professions Council (HCPC) data on registered UK radiographers were not used to determine the radiographer population size due to potential inconsistencies, as some registrants have left the UK or have not yet been employed in the UK. Therefore, the sample size was calculated using the formula for large, unknown populations, yielding an adequate sample size for estimating the prevalence of workplace bullying and harassment within the desired margin of error.

The study sample substantially exceeded the recommended minimum sample sizes for multivariable regression analyses. The initial target of 384 participants was derived from Cochran's formula (14) for estimating a population proportion with 95% confidence and a ± 5% margin of error. This calculation supported the descriptive survey objective and did not guarantee statistical significance. Because the principal analyses involved multivariable regression and exploratory indirect-effect estimation, a sensitivity analysis of the achieved sample was subsequently undertaken to characterise the inferential precision supported by the final analytical sample.

The fullest adjusted model included 10 predictors and 357 complete observations. At a two-sided *α* of 0.05, this sample provided 80% power to detect an overall multiple-regression effect of approximately Cohen's $f^{2}=0.048\;$ and 90% power to detect an effect of approximately $f^{2}=0.061$. Thus, the achieved sample size was sufficient to detect associations larger than a conventional small effect, although it may not have identified very small associations (15). The exploratory indirect statistical association was evaluated using 5,000 bootstrap samples because the sampling distribution of a product term is often non-normal. Because mediation power depends on the joint sizes of the constituent paths, the bootstrap confidence interval was used to assess the precision of the indirect association, and no causal or confirmatory mediation claim was made (16). The final sample was 385.

### Participants and eligibility criteria

Participants were HCPC-registered diagnostic or therapeutic radiographers currently practising in the United Kingdom. Eligible participants were required to be actively practising at the time of survey completion. Students and individuals not practising in the UK were excluded.

### Recruitment and data collection

Participants were recruited via professional networks, professional societies, and digital professional platforms. This approach is widely used in occupational workforce research where comprehensive sampling frames are not readily accessible (17). The survey was administered electronically. Participation was voluntary and anonymous. Participants provided informed consent before participating.

### Data collection procedure

A team of seven (7) academic and clinical radiographers based in different regions of the UK developed the survey, which was administered through JISC Online Surveys. The questionnaire consisted of 35 questions, with a mix of closed-ended responses and free-text responses, to explore demographic information and experiences of workplace bullying and harassment. The online survey was open from 18th December 2025 to 3rd February 2026 and was advertised through professional societies, interest groups, social media, and LinkedIn, with online reminders. Results were analysed in SPSS version 27.

The online questionnaire incorporated the validated NAQ-R alongside study-specific Speak-Up Climate and Safety Impact items. Cognitive pretesting and pilot testing assessed clarity and contextual relevance but did not formally validate the study-specific composites. Participants provided consent in the preliminary section of the survey form, which included their information, to ensure full comprehension of the study before accessing the survey. Survey responses were anonymous and stored securely. The submission package includes the questionnaire and all supplementary tables and figures.

### Cognitive pretesting and pilot testing

Cognitive pre-testing was conducted with 15 radiographers to assess the comprehension and appropriateness of the questions. A pilot study among 10 radiographers was also conducted, during which the survey questions were revised for clarity and relevance to the UK context.

### Measures and instrumentation

Workplace bullying exposure was measured using the Negative Acts Questionnaire–Revised (*N*AQ-R), a validated behavioural instrument assessing exposure to bullying behaviours over the preceding six months (18). The NAQ-R includes 22 items scored on a five-point frequency scale ranging from never to daily. The NAQ-R has demonstrated strong reliability and validity across occupational and healthcare populations (18).

The speak-up climate was assessed using a composite scale comprising four Likert-type items assessing psychological safety, fear of negative consequences when raising concerns, and confidence in the organisational response. Higher scores indicated a poorer speak-up climate. These constructs are consistent with established psychological safety and organisational voice frameworks (1, 18).

Perceived compromise in patient safety was assessed using a composite scale comprising six Likert-type items measuring perceived risk to patient safety, disruption to teamwork, impaired adherence to protocols, and diminished confidence in safe practice. Covariates included age, gender, ethnicity, years of experience, job band, employment setting, modality, and country of professional training. The composite measures were developed from established theoretical constructs related to psychological safety, organisational voice, and perceptions of patient safety culture (1, 2, 13).

### Statistical analysis

Data were analysed using SPSS version 27. Descriptive statistics summarised participant characteristics and the prevalence of WBH exposures. Questions permitting multiple selections were analysed as multiple-response items. Each response option was reported as the number and percentage of eligible participants selecting that option, using the total study sample (*N* = 385) as the denominator. Because participants could select more than one option, percentages may exceed 100%. The total number of selections was reported separately but was not used as the denominator for participant-level percentages.

Multivariable linear regression models examined associations between exposure to workplace bullying, speak-up climate score, and perceived compromise of patient safety. Sequential models assessed attenuation of associations after adjustment for speak-up climate score. Internal consistency was evaluated using Cronbach's alpha, corrected item-total correlations, mean inter-item correlations, and first-component variance. Associations were estimated using Spearman correlations with 95% confidence intervals and Benjamini-Hochberg false-discovery-rate-adjusted q-values for the correlation families.

The Safety Impact Score and Speak-Up Climate Score were analysed as continuous variables because each represented the mean of multiple five-point Likert items measuring a common construct. Composite scores derived from multiple Likert items are commonly treated as approximately continuous in linear regression analyses when they demonstrate acceptable internal consistency and approximately symmetric score distributions (19). The primary regression analyses used valid person-mean-prorated composite scores and complete data for the model covariates. Separate sensitivity analyses required complete responses to every constituent scale item and every included covariate. Inference used HC3 heteroscedasticity-consistent standard errors. Model assumptions were evaluated by examining residual-versus-fitted plots, normal Q-Q plots, Cook's distance and variance inflation factors (VIFs).

Adjusted linear regression models used heteroscedasticity-consistent HC3 standard errors. Covariates were age, gender, ethnicity indicators, UK/non-UK training, NHS/non-NHS setting, years of experience and job band. Exploratory mediation was estimated using 5,000 bootstrap samples.

#### Item development

The seven-member research team developed the Safety Impact Score and Speak-Up Climate Score items *a priori* after reviewing the literature on psychological safety, employee voice and silence, psychosocial safety climate, and patient safety culture. The Safety Impact items were mapped to perceived patient-safety risk, teamwork disruption, protocol adherence and confidence in safe practice. The Speak-Up Climate items were mapped to anticipated negative consequences of speaking up, perceived difficulty raising patient-care concerns, hesitation to report bullying or safety incidents, and confidence in organisational responsiveness. Cognitive pretesting with 15 radiographers assessed interpretation and comprehension, and pilot testing with 10 radiographers assessed clarity, relevance and response-option suitability.

#### Dimensionality analysis

The preliminary internal structure was examined using both principal component analysis and principal axis factoring. PCA described the first eigenvalue of the correlation matrix, the percentage of total item variance explained by the first component, and the component loadings. Principal axis factoring estimated the one-factor common-variance solution, including the extraction eigenvalue, unrotated factor loadings, and extracted communalities. Factorability was evaluated using the Kaiser–Meyer–Olkin statistic and Bartlett's test of sphericity. Analyses used complete item responses, and Q33_4 was reverse-coded before analysis. These analyses were regarded as preliminary evidence of internal structure rather than confirmation of construct validity.

To assess whether the two study-specific composites represented related but distinct constructs, their inter-scale correlation was examined alongside separate principal component and principal axis factor analyses. Because the measures were exploratory rather than established latent instruments, these analyses were treated as preliminary evidence of construct distinctiveness rather than formal validation.

### Scoring template

Exposure to workplace bullying and harassment was operationalised as the mean of the 22 NAQ-R items (Q13_1 to Q13_22), with a prorated total score also reported. The Safety Impact Score was calculated as the mean of F1-F6 (Q32_1 to Q32_6), with a minimum of 5 valid items. The Speak-Up Climate Score was calculated as the mean of F7-F10 (Q33_1 to Q33_4), with a minimum of 3 valid items.

Positively worded items were reverse—coded prior to composite score construction to ensure directional consistency across all scale items (20). Because F10 is positively framed (‘*I feel confident that concerns raised in my department are addressed fairly and promptly’*), it was reverse-coded prior to averaging to ensure conceptual consistency across mixed-worded scales (21). Higher scores, therefore, indicated a poorer speak-up climate. F11 (Q34_1) was retained as a single-item indicator of perceived malicious or vexatious use of Health and Care Professions Council (HCPC) referral processes. Q1_1 to Q34_1 are shown in supplementary Appendix S1 (study questionnaire).

### Missing data handling

Missing data were handled using person-mean prorating (22) rather than traditional mean substitution. Composite scores were calculated only when respondents completed at least five of the six Safety Impact items or three of the four Speak-Up Climate items, corresponding to approximately 75%–80% completion of the constituent items. Requiring completion of at least five of six or three of four items ensured that composite scores reflected most of the intended construct while avoiding unnecessary exclusion due to isolated missing responses (22).

For eligible respondents, the composite score was calculated as the arithmetic mean of the completed items. Participants who did not meet these minimum completion thresholds were assigned missing values for the composite score and were excluded from analyses involving that outcome. As a sensitivity analysis, the principal regression models were repeated using only respondents with complete data on every item contributing to the variables included in each model. The number of observations included in each analysis is therefore reported for transparency.

### Complete-case sensitivity analysis

Sensitivity analyses were repeated using participants with complete responses to every item contributing to the NAQ-R, Safety Impact and Speak-Up Climate measures required for the relevant model, together with complete data for all included covariates. The NAQ-R was retained as a mean score on its original 1–5 response scale, consistent with the primary analyses. The complete-case models used the same covariate specification and HC3 heteroscedasticity-consistent standard errors as the primary models. An additional exploratory indirect-association analysis was conducted in the common complete-case sample using 5,000 bootstrap resamples.

### Procedural consideration of common-method bias

Because the principal constructs were measured using the same self-report questionnaire, several procedural steps were used that may reduce common-method bias. Participation was anonymous and voluntary, questions relating to the principal constructs were organised into separate sections, and the questionnaire incorporated both positively and negatively framed items. Cognitive pretesting and pilot testing were undertaken to improve item clarity and reduce ambiguity. These procedures may reduce evaluation apprehension, demand characteristics and mechanically consistent responding, but cannot remove bias arising from same-source, contemporaneous measurement (23).

### Qualitative free-text analysis

Responses to the optional open-ended question were analysed using conventional qualitative content analysis (24). Respondents provided optional free-text comments in response to the question: “*Please feel free to suggest ways your organisation could better prevent bullying or support staff facing bullying.”* A total of 188 respondents provided analysable free-text responses. This approach was selected because the free-text responses were intended to provide descriptive insight into participants’ recommendations for preventing and managing workplace bullying and harassment, thereby complementing the quantitative findings.

Two researchers (CO and EI) independently coded all 188 responses using the initial codebook. Coding outputs were compared, disagreements were discussed item by item and resolved by consensus, and the codebook was revised iteratively before final category development. The wider research team (NJ, RE, BNE, RM, OL) reviewed the evolving codebook, category structure and illustrative extracts and challenged interpretations where necessary but did not independently recode all responses. Reflexive notes documented coding decisions, changes to category definitions and researcher assumptions. The analysis followed conventional qualitative content analysis and was intended to produce descriptive categories rather than an interpretive thematic account (24). Representative illustrative quotations are presented using anonymised participant identifiers such as P001 and P002 to preserve confidentiality while enhancing analytical transparency.

Given that the qualitative data were derived from optional survey free-text responses rather than in-depth interviews, the analysis was intended to provide contextual and explanatory insight into the quantitative findings rather than a fully interpretive phenomenological exploration. The qualitative findings were therefore integrated with the quantitative results to support complementary qualitative insights and enrich understanding of organisational barriers to speaking up, psychological safety, and WBH prevention within UK radiography practice (24, 25).

### Ethical considerations

The Health Sciences University Research Ethics Committee approved the study (HRS-2025-YHAiR). Participation was voluntary and anonymous, and the study followed the principles outlined in the Declaration of Helsinki. Signposting to support was provided despite the minimal risk of possible distress from recalling adverse events. Consent was obtained at the beginning of the survey.

## Result

### Participant demographics and professional characteristics

The average age of participants was approximately 40 years (SD = 8.4), with most respondents female, NHS-employed, Band 6-7, aged 35-44 years (54.3%; n = 209), and working across sonography, CT, projectional radiography, and MRI. The largest group identified as Black, Black British, Caribbean, or African (61.8%; n = 238) and more than half of respondents (55.1%; n = 212) received their primary radiography education outside the United Kingdom. See Table 1 for more details.

**Table 1:** Participant demographics data (*N* = 385).

Demographics		Frequency	Percentage
Age	Under 25	5	1.3
	25–34	90	23.4
	35–44	209	54.3
	45–54	65	16.9
	55–64	16	4.2
Gender	Male	182	47.3
	Female	201	52.2
	Non-binary	1	0.3
	Prefer not to say	1	0.3
Ethnicity	Asian/Asian British	12	3.1
	Black, Black British, Caribbean or African	238	61.8
	Mixed/Multiple ethnic groups	8	2.1
	White	120	31.2
	Other ethnic group	7	1.8
Country of Radiography Education	UK	173	44.9
	Non-UK	212	55.1
Primary Employment setting	NHS Trust	321	83.4
	Private Hospital/Clinic	29	7.5
	University/Academic	11	2.9
	Locum/Agency	14	3.6
	Other/missing (please specify)	10	2.6
Years of Experience	Less than 1 year	4	1.0
	1–5 years	52	13.5
	6–10 years	91	23.6
	11–15 years	119	30.9
	16–20 years	54	14.0
	Over 20 years	61	15.8
	Missing	4	1.0
Current Job Level	Band 5	28	7.3
	Band 6	132	34.3
	Band 7	157	40.8
	Band 8 or above	52	13.5
	Non-AFC role	13	3.4
	Other (please specify)	3	0.8
Country of Practice	England	358	93.0
	Scotland	16	4.2
	Wales	4	1.0
	Northern Ireland	5	1.3
	Missing	2	0.6
Employment pattern	Full-time	302	78.4
	Part-time	64	16.6
	Bank/Locum	16	4.2
	In-sourcing companies	3	0.8
Primary role (multiple responses, *n* = 495)	x-ray/Projectional radiography	77	15.6
	CT	91	18.4
	MRI	76	15.4
	Mammography	11	2.2
	Nuclear Medicine	5	1.0
	Therapeutic Radiographer	34	6.9
	Sonographer	120	24.2
	Managers	14	2.8
	Practice Educator	11	2.2
	DEXA	5	1.0
	Reporting Radiographer	23	4.6
	Other (please specify)	28	5.7

‘n’ for the multiple-response primary-role item percentages is based on 495 selections/denominator rather than 385 participants.

#### Preliminary psychometric evaluation of the study-specific composite measures

A total of 385 UK-practising radiographers were included in the analysis. The NAQ-R bullying exposure scale showed excellent internal consistency (Cronbach's alpha = 0.94), as did the Safety Impact Score derived from F1-F6 (alpha = 0.90). The Speak-Up Climate Score, calculated from F7-F10 after reverse-coding F10, demonstrated borderline internal consistency (alpha = 0.67), as shown in Table 2. Valid mean scores were available for 381 participants on the NAQ-R, 372 on Safety Impact, and 384 on Speak-Up Climate. Table 3 and Figure 1 provide details.

**Figure 1:**
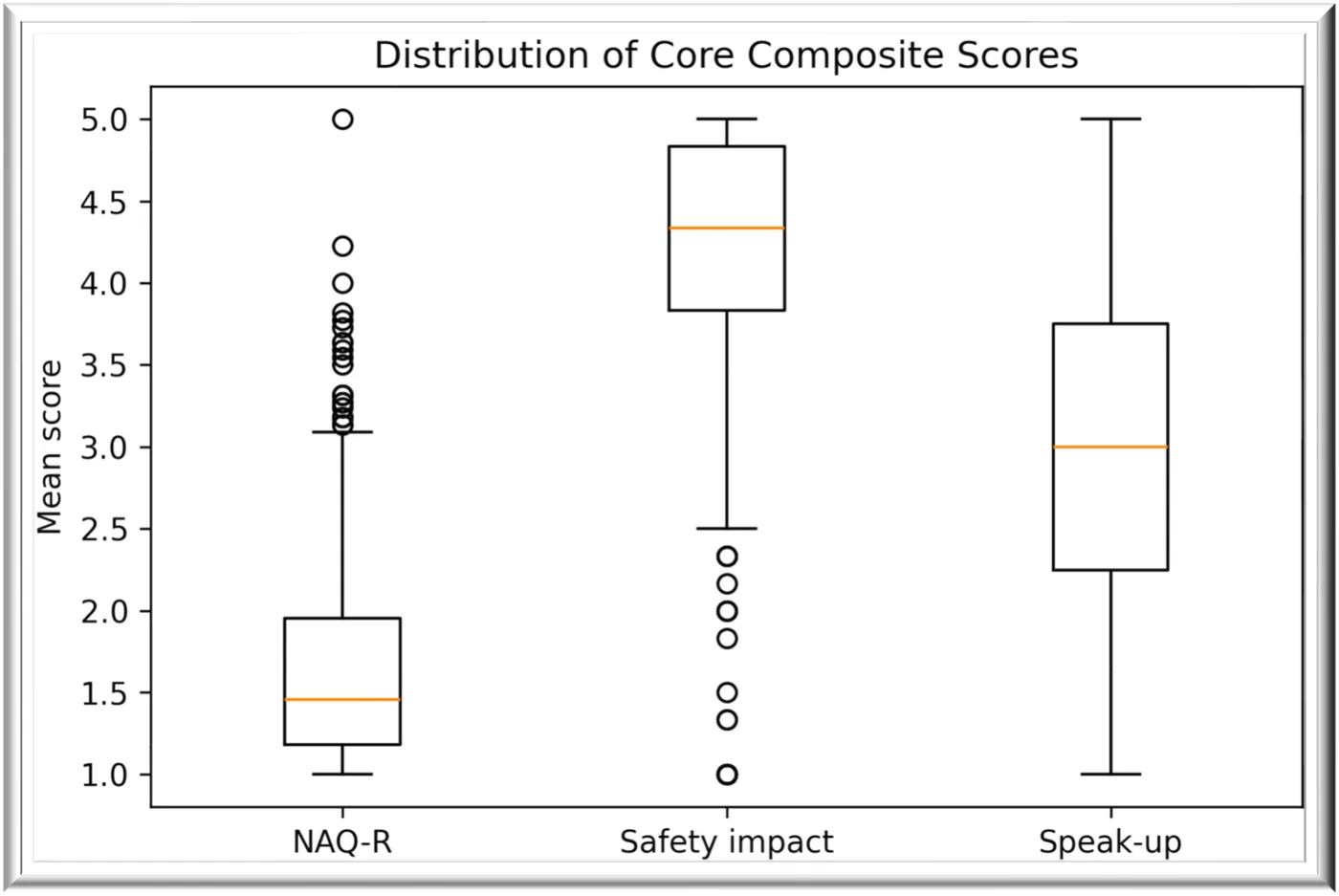
Boxplot of core composite scores.

**Table 2:** Reliability and dimensionality summary for NAQ-R, safety impact and speak-up climate scales.

Scale	Items	Complete (n)	Cronbach's alpha	Mean inter-item (r)	First eigenvalue	Variance explained by first component (%)
NAQ-R bullying exposure	22	365	0.94	0.43	10.18	46.14
Safety Impact Score	6	366	0.90	0.61	4.08	67.74
Speak-Up Climate Score	4	383	0.67	0.33	2.05	51.15

**Table 3:** Descriptive statistics for core composite and single-item scores.

Score	n	Mean	SD	Median	IQR	Min	Max	Missing
NAQ-R mean	381	1.66	0.66	1.45	0.77	1.00	5.00	4
NAQ-R sum	381	36.57	14.62	32.00	17.00	22.00	110.00	4
Safety Impact Score	372	4.15	0.96	4.33	1.00	1.00	5.00	6
Speak-Up Climate Score	384	3.01	0.97	3.00	1.50	1.00	5.00	1
F11 malicious/vexatious HCPC concern	385	3.49	1.21	3.00	2.00	1.00	5.00	0

For the Safety Impact Score, 366 participants provided complete item responses. The correlation matrix demonstrated good factorability (KMO = 0.870; Bartlett's *χ*^2^ (15) = 1428.26, *p* < 0.001). PCA produced a first-component eigenvalue of 4.064, representing 67.74% of total item variance, with component loadings from 0.649 to 0.884. The one-factor principal axis extraction eigenvalue was 3.714, representing 61.90% of total item variance; factor loadings ranged from 0.565 to 0.871 and communalities from 0.319 to 0.758. Details are in Tables 2, 4, 5.

**Table 4:** Preliminary dimensionality assessment .

Composite measure	KMO	Bartlett's test	First eigenvalue	Variance explained
Safety impact score	0.87	*χ*^2^ (15) = 1428.26, *p* < 0.001	4.08	67.74%
Speak-up climate score	0.68	χ^2^ (6) = 268.87, *p* < 0.001	2.05	51.15%

**Table 5:** Complete item-level matrix dimensionality.

Measure/item	PCA loading	PAF loading	PAF communality
Safety Q32_1	0.649	0.565	0.319
Safety Q32_2	0.816	0.767	0.588
Safety Q32_3	0.854	0.823	0.677
Safety Q32_4	0.835	0.793	0.629
Safety Q32_5	0.878	0.862	0.743
Safety Q32_6	0.884	0.871	0.758
Speak-up Q33_1	0.712	0.549	0.301
Speak-up Q33_2	0.770	0.629	0.395
Speak-up Q33_3	0.849	0.866	0.749
Speak-up Q33_4 reversed	0.473	0.312	0.097

For Speak-Up Climate, complete item responses were available for 383 participants. Factorability was more modest but adequate (KMO = 0.678; Bartlett's *χ*^2^ (6) = 268.87, *p* < 0.001). PCA produced a first-component eigenvalue of 2.046, representing 51.15% of total item variance, with component loadings from 0.473 to 0.849. The one-factor principal axis extraction eigenvalue was 1.543, representing 38.59% of total item variance; factor loadings ranged from 0.312 to 0.866 and communalities from 0.097 to 0.749. The reverse-coded organisational-response item had the weakest loading (0.312) and communality (0.097), supporting cautious interpretation of the composite.

Corrected item–total correlations for the six-item Safety Impact Score ranged from 0.54 to 0.80, indicating that all constituent items contributed meaningfully to the composite measure. For the four-item Speak-Up Climate Score, corrected item–total correlations ranged from 0.26 to 0.63. Although the reverse-coded item assessing organisational responsiveness had a lower item–total correlation than the others, we retained it for its theoretical relevance to psychological safety and organisational voice. Details are in S2.

### Discriminant validity between the speak-up climate score and safety impact score

Although the two study-specific composites were moderately correlated (Spearman *ρ* = 0.33), joint dimensionality analyses supported their empirical distinction. Principal axis factoring identified two correlated dimensions corresponding to Speak-Up Climate and Safety Impact, with items loading primarily on their intended construct and no substantial cross-loadings. These findings indicate that the two composites represent related but distinguishable organisational constructs rather than a single underlying dimension, while recognising that same-source measurement may still contribute to shared variance.

### Preliminary dimensionality

Complete item data were available for 366 participants for Safety Impact and 383 for Speak-Up Climate. The Safety Impact correlation matrix showed good factorability (KMO = .870; Bartlett's *χ*^2^ (15) = 1428.26, *p* < 0.001). PCA yielded a first-component eigenvalue of 4.064, accounting for 67.74% of total item variance; component loadings ranged from 0.649 to 0.884. In the one-factor principal axis solution, the extraction eigenvalue was 3.714, accounting for 61.90% of total item variance. PAF loadings ranged from 0.565 to 0.871, and communalities ranged from 0.319 to 0.758, providing preliminary support for a dominant common dimension.

The Speak-Up Climate matrix showed more modest factorability (KMO = 0.678; Bartlett's *χ*^2^ (6) = 268.87, *p* < 0.001). PCA yielded a first-component eigenvalue of 2.046, accounting for 51.15% of total item variance; component loadings ranged from 0.473 to 0.849. The one-factor PAF extraction eigenvalue was 1.543, accounting for 38.59% of total item variance. PAF loadings ranged from 0.312 to 0.866, and communalities ranged from 0.097 to 0.749. Q33_4 had the weakest PAF loading (0.312) and communality (0.097), supporting cautious interpretation of the four-item composite. Details are in Tables 4–6.

**Table 6:** Principal axis factoring variables.

Scale	PAF extraction eigenvalue	Percentage of total item variance represented by extracted factor	PAF loading range	Extracted communality range
Safety impact	3.714	61.90%	0.565–0.871	0.319–0.758
Speak-up climate	1.543	38.59%	0.312–0.866	0.097–0.749

### Organisational safety and speak-up climate

The formal-reporting questions, “Did you formally report the bullying and harassment…?” and “If you did not report the bullying/harassment or delayed reporting, what were the reason(s)?”, allowed multiple selections. Of the 385 participants, 373 selected at least one option, generating 430 selections. Reporting to a line manager was selected by 118 participants (30.6%), HR by 32 (8.3%), local incident-reporting or speaking-up channels by 27 (7.0%), the Society of Radiographers or another trade union by 15 (3.9%), and the HCPC by one participant (0.3%). A total of 130 participants (33.8%) selected that they had not formally reported the behaviour, while 107 (27.8%) selected “not applicable.” Categories were not mutually exclusive. Figure 2 shows the distribution of Speak-Up Climate and HCPC Referral Perception items.

**Figure 2:**
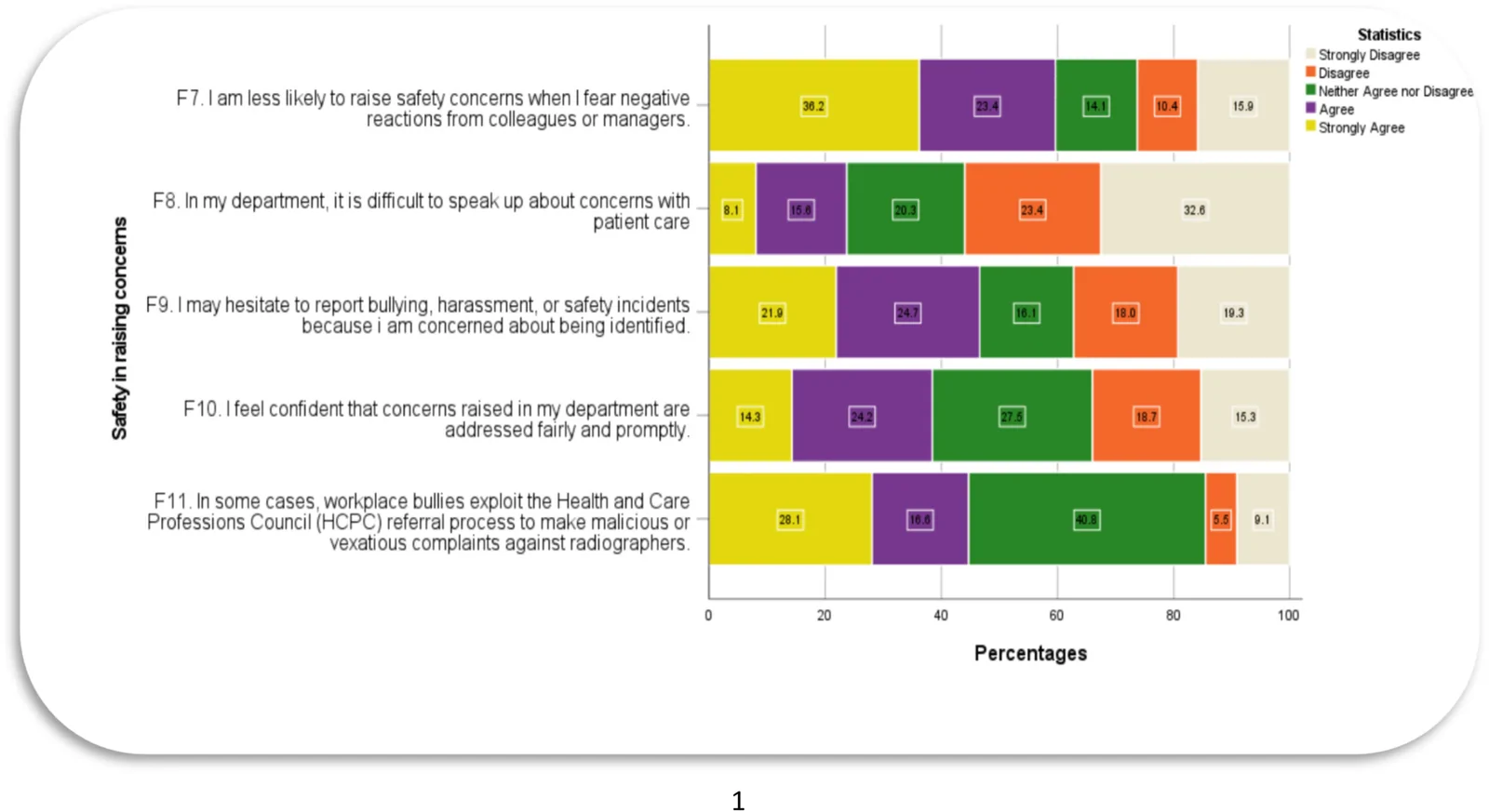
Distribution of Speak-Up Climate and HCPC Referral Perception Items.

All 385 participants responded to the multiple-response question on reasons for non-reporting or delayed reporting, yielding 659 selections. The most commonly cited reasons were lack of trust in the reporting system (107 participants, 27.8%), the belief that nothing would change (100 participants, 26.0%), and fear of retaliation (90 participants, 23.4%). Other reasons included not recognising the behaviour as bullying at the time (64, 16.6%), considering it “part of the job” (35, 9.1%), competing work demands (33, 8.6%), and uncertainty about how to report (23, 6.0%). Details are in Table 7.

**Table 7:** Reporting behaviours, barriers to reporting and perceived organisational response.

Questions	Options	n	%
Did you formally report the bullying/harassment? (multiple responses, *n* = 430) *N* = 385	Yes, to HR	32	8.3
	Yes, to the line manager	118	30.6
	Yes, HCPC	1	0.3
	Yes, SoR or other trade union	15	3.9
	Yes, via local incident reporting channels (e.g., Datix, freedom to speak up guardian, whistleblowing)	27	7.0
	No	130	33.8
	Not Applicable	107	27.8
If you did not report the bullying/harassment or delayed reporting, what were the reason (s)? (multiple responses, *n* = 659)	Fear of retaliation	90	23.4
	Lack of trust in the reporting system	107	27.8
	Thought nothing would change	100	26.0
	Didn't recognise it as bullying at the time	64	16.6
	Did not know how to report	23	6.0
	Too busy with work and other things	33	8.6
	Considered it as “part of the job"	35	9.1
	Not Applicable	195	50.6
	Yes, other (please specify details)	12	3.1
If reported, was the matter addressed satisfactorily?	Yes	23	6.0
	No	67	17.4
	Partially	34	8.8
	Still ongoing	12	3.1
	Don't know/not informed of outcome	31	8.1
	Not Applicable	216	56.1
	Other (please specify)	2	0.5

Participants could select more than one option. Percentages use the total eligible sample (*N* = 385) as the denominator and therefore do not sum to 100%. Participants generated multiple selections (n): 430 selections for reporting routes and 659 selections for reasons for non-reporting or delayed reporting.

### Correlation patterns with patient safety and professional impact outcomes

NAQ-R bullying exposure was positively associated with the Safety Impact Score (Spearman's rho=0.17, 95% CI 0.07 to 0.27, *p* = 0.003). Item-level correlations between NAQ-R and F1-F6 were small but generally statistically significant, except for F5. By contrast, associations between NAQ-R exposure and speak-up climate indicators were stronger. NAQ-R was associated with F7 (rho=0.21), F8 (rho=0.43), F9 (rho=0.43), reverse-coded F10 (rho=0.36), F11 (rho=0.18) and the overall Speak-Up Climate Score (rho=0.51; all q < 0.001 except F11, q = 0.001). Details are in Tables 8, 9 and Figures 3, 4.

**Table 8:** Spearman correlations between NAQ-R bullying exposure and patient safety impact items.

Outcome	N	Spearman rho	95% CI lower	95% CI upper	p	q (BH-FDR)
F1	381	0.17	0.07	0.27	<0.001	0.003
F2	380	0.13	0.03	0.23	0.010	0.011
F3	381	0.15	0.05	0.24	0.004	0.006
F4	378	0.19	0.09	0.29	<0.001	0.001
F5	368	0.10	−0.01	0.20	0.063	0.063
F6	368	0.15	0.05	0.25	0.004	0.006
Safety Impact Score	369	0.17	0.07	0.26	0.001	0.003

**Table 9:** Spearman correlations between NAQ-R bullying exposure and speak-up climate indicators.

Outcome	n	Spearman rho	95% CI lower	95% CI upper	p	q (BH-FDR)
F7	380	0.21	0.11	0.31	<0.001	<0.001
F8	380	0.43	0.35	0.51	<0.001	<0.001
F9	380	0.43	0.35	0.51	<0.001	<0.001
F10 reverse-coded	381	0.36	0.27	0.45	<0.001	<0.001
F11	381	0.18	0.08	0.27	<0.001	<0.001
Speak-Up Climate Score	380	0.51	0.43	0.58	<0.001	<0.001

**Figure 3:**
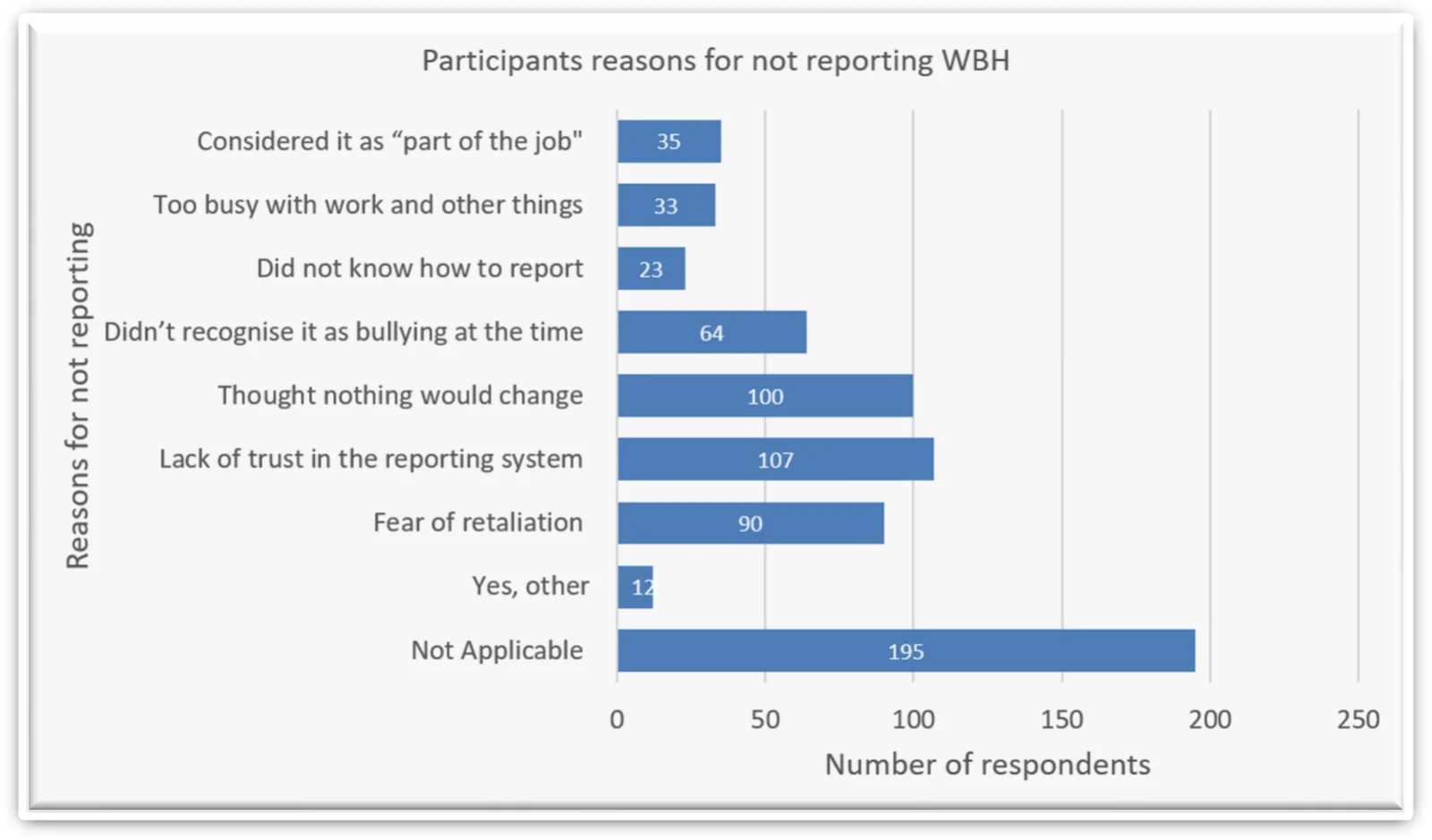
Participants' reasons for not reporting or delaying reporting of workplace bullying and harassment.

**Figure 4:**
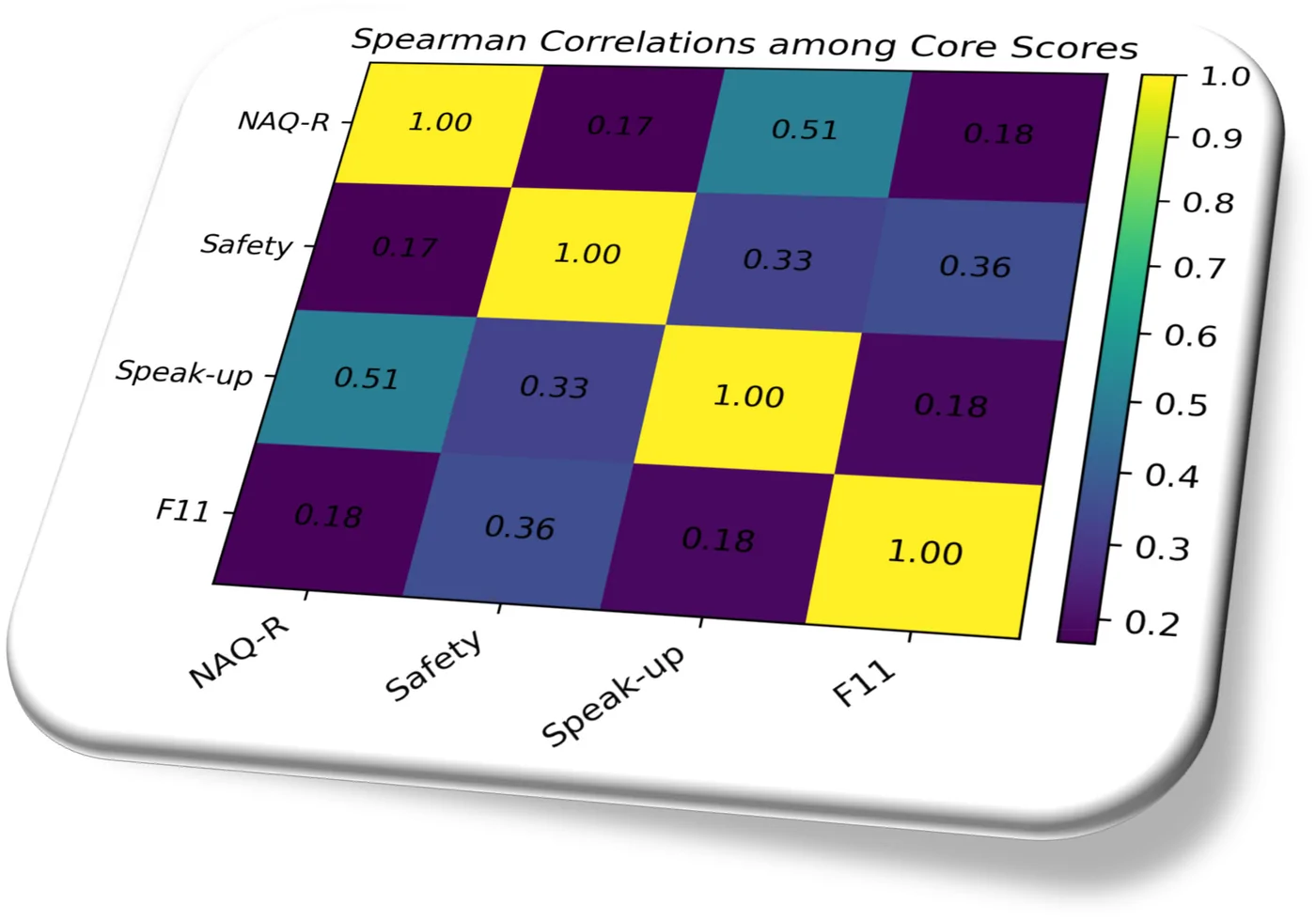
Spearman correlation heatmap for NAQ-R, Safety Impact, Speak-Up Climate and F11. The heatmap illustrates the strength and direction of Spearman correlations between workplace bullying, speak-up climate, and patient safety-related variables. Darker colours indicate stronger associations. Workplace bullying demonstrated the strongest relationships with Speak-Up Climate indicators. F11 refers to the item addressing malicious reporting by WBH perpetrators.

Multicollinearity was low for the focal predictors. The maximum VIF was 4.69, below the commonly used threshold of 5. No observation was automatically excluded as influential, although the maximum Cook's distances varied across models. Residual-versus-fitted plots did not indicate substantial non-linearity. Because residual distributions departed from normality, we used HC3 heteroscedasticity-consistent standard errors.

### Adjusted regression

Adjusted regression models showed that NAQ-R exposure was independently associated with poorer Speak-Up Climate Score (B = 0.688, 95% CI 0.550 to 0.825, *p* < 0.001) and with higher Safety Impact Score before adjustment for Speak-Up Climate (B = 0.316, 95% CI 0.179 to 0.453, *p* < 0.001). After the Speak-Up Climate Score was added to the safety model, the NAQ-R coefficient attenuated markedly and was no longer statistically significant (B = 0.052, 95% CI −0.095 to 0.199, *p* = 0.488), whereas Speak-Up Climate Score remained independently associated with Safety Impact Score (B = 0.378, 95% CI 0.255 to 0.501, *p* < 0.001). Details are in Table 10 and Figure 5.

**Table 10:** Summary of adjusted regression models.

Model	Outcome	N	Key predictor	B	95% CI	p	R^2^/adjusted R^2^
1	Poorer Speak-Up Climate	369	NAQ-R mean	0.688	0.550 to 0.825	<0.001	0.250/0.231
2	Perceived Safety Impact	358	NAQ-R mean	0.316	0.179 to 0.453	<0.001	0.118/0.096
3	Perceived Safety Impact	357	NAQ-R mean	0.052	−0.095 to 0.199	0.488	0.230/0.208
3	Perceived Safety Impact	357	Speak-Up Climate	0.378	0.255 to 0.501	<0.001	0.230/.208

All models used ordinary least squares regression with HC3 heteroscedasticity-consistent standard errors.

**Figure 5:**
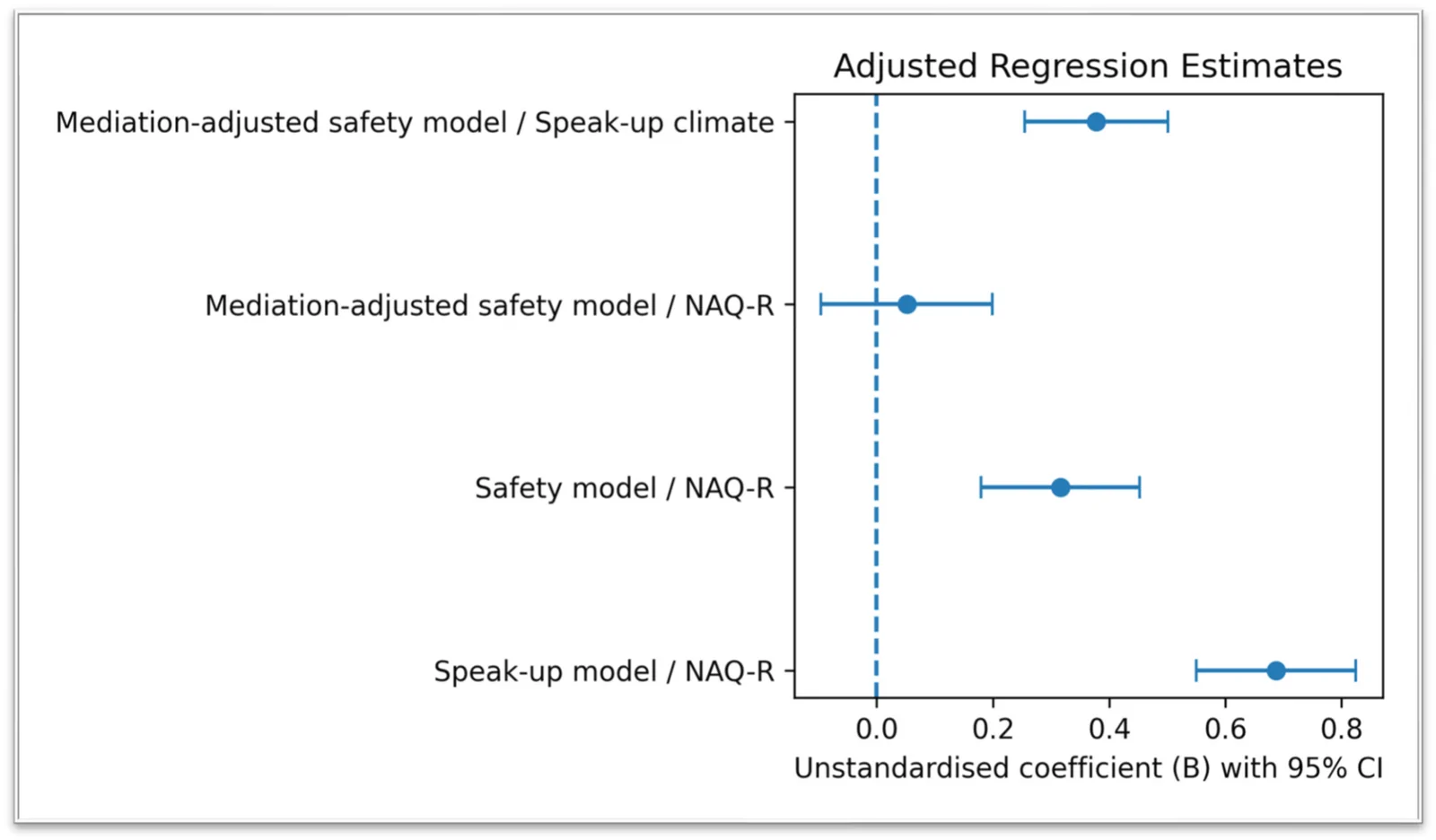
Forest plot of adjusted regression estimates for primary predictors. Forest plots display adjusted regression coefficients and 95% confidence intervals. Values to the right of zero indicate a positive association with the outcome variable. Confidence intervals crossing zero indicate non-significant associations.

### Mediation analysis

An exploratory mediation analysis was conducted to assess whether the Speak-Up Climate statistically accounted for the observed association between workplace bullying and perceived compromise of patient safety. Given the cross-sectional study design, the mediation analysis was interpreted as assessing indirect statistical associations rather than causal statistical relationships.

The bootstrapped indirect association was 0.264 (95% CI 0.169 to 0.373). The observed pattern was consistent with an indirect association involving Speak-Up Climate; however, the cross-sectional design does not establish temporal ordering or a causal mechanism. This interpretation aligns with psychological safety theory and employee voice/silence frameworks, but causal claims are not warranted given the cross-sectional nature of the data (1, 27, 28). Details are provided in Supplementary Table S3 and Figure 6.

**Figure 6:**
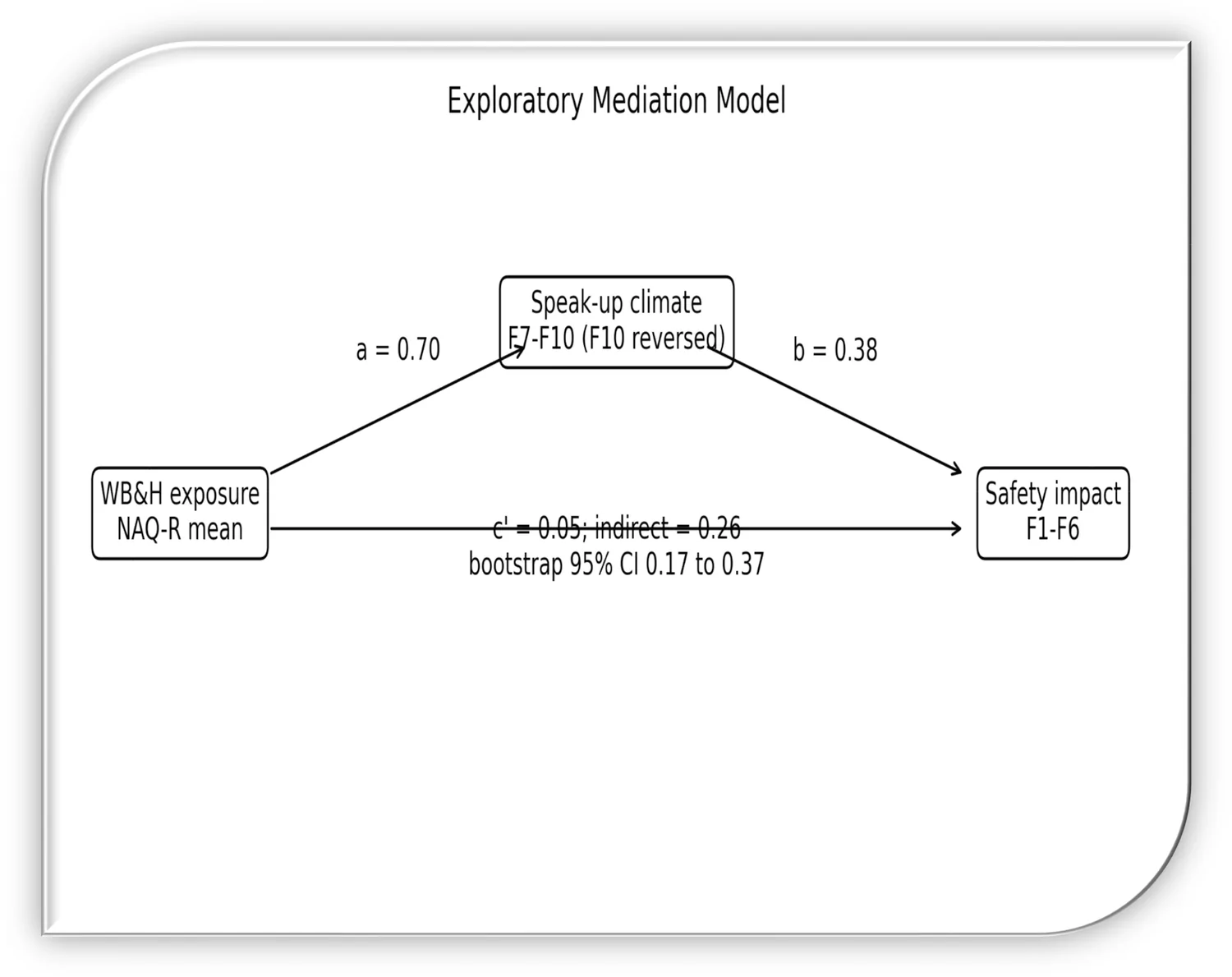
Exploratory mediation model. The mediation model illustrates the hypothesised indirect statistical association linking exposure to workplace bullying to perceived compromise of patient safety involving speak-up climate. Unstandardized coefficients are shown. As the data are cross-sectional, the model should be interpreted as exploratory rather than as evidence of causality. The arrows represent the statistical model rather than established temporal direction.

### Missing data analyses

Missingness was limited across the composite measures. Safety Impact item-level missingness ranged from 0% to 3.6%; 366 participants completed all six items, six (1.6%) required person-mean prorating, and 13 (3.4%) did not meet the five-item threshold. Speak-Up Climate item-level missingness ranged from 0% to 0.3%; 383 participants completed all four items, one (0.3%) required prorating, and one (0.3%) did not meet the three-item threshold. Details are in Supplementary Table S4.

### Sensitivity analyses

Sensitivity analyses supported the robustness of the primary findings. The adjusted models were re-estimated using three restricted samples: NHS-employed respondents only, UK-trained respondents only, and diagnostic-only respondents. Across all three analyses, NAQ-R bullying exposure remained strongly associated with poorer Speak-Up Climate, whereas the direct association between bullying exposure and Safety Impact became non-significant after adjustment for Speak-Up Climate. Bootstrapped indirect associations remained positive across all sensitivity samples. Details are provided in Supplementary Tables S5–S6.

These findings strengthen the interpretation that impaired psychological safety and a reduced speak-up climate statistically account for the association between WBH and perceived safety impact, rather than a simple direct association. However, given the cross-sectional design, these findings should be interpreted as consistent with an indirect association rather than proof of causal mediation, in line with methodological cautions around mediation analysis in non-longitudinal data (31).

#### Complete-case sensitivity analyses

Complete−case analyses yielded estimates materially similar to those of the primary analyses. NAQ−R exposure remained associated with poorer Speak−Up Climate (*N* = 353; B = 0.686, 95% CI 0.546–0.825, *p* < 0.001) and with greater perceived Safety Impact before adjustment for Speak−Up Climate (*N* = 337; B = 0.305, 95% CI 0.167–0.442, *p* < 0.001). After Speak−Up Climate was included, the direct NAQ−R association remained non−significant (B = 0.054, 95% CI −0.098–0.207, *p* = .487), while Speak−Up Climate remained associated with perceived Safety Impact (B = 0.366, 95% CI 0.238–0.494, *p* < .001). The indirect association was also similar (B = 0.250, 95% bootstrap CI 0.154–0.364). Full comparisons with the primary analyses are provided in Supplementary Tables S7–S9.

### Qualitative findings from free-text responses

The free-text responses provide descriptive contextualisation of the quantitative findings and will inform the design of the subsequent in-depth qualitative interview phase for the next study to be reported separately. Free-text responses provided additional insight into respondents’ perceptions of organisational contributors to workplace bullying, as well as potential strategies for prevention and staff support. Several recurring content categories emerged, including organisational silence, psychological unsafety, and building psychologically safe workplace cultures. Overall, respondents frequently described workplace bullying as a systemic organisational issue rather than solely an interpersonal conflict. Many participants expressed concerns that existing reporting structures lacked confidentiality, independence, or meaningful follow-up, contributing to reluctance to raise concerns formally.

### Category 1: organisational silence and psychological unsafety

Participants frequently described workplace environments where fear of retaliation, victimisation, or reputational harm deterred staff from speaking up about bullying, harassment, or patient safety concerns. Many respondents feared that reporting inappropriate behaviour could lead to workplace social and professional isolation, managerial backlash, career disadvantage, or being labelled as ‘problematic’ within departments. These responses reflected broader organisational dynamics of silence and diminished psychological safety within imaging workplaces.

Several respondents specifically emphasised a reluctance to raise concerns for fear of being identified, particularly in smaller departments where anonymity was perceived to be difficult to maintain. Others described low confidence in existing reporting systems, expressing concerns that complaints were often ignored, minimised, or inadequately investigated. Collectively, these comments suggested that staff frequently perceived speaking up as professionally risky.

Illustrative comments included:

“People are scared to report because everyone knows who raised the concern.” (P117)

“There is fear that speaking up will make you a target.” (P203)

“Bullying gets ignored if the person involved is senior or well-connected.” (P061)

### Category 2: leadership and organisational accountability

Participants repeatedly highlighted the perceived importance of leadership behaviours, managerial accountability, and organisational responsiveness in shaping workplace culture. Many respondents described frustrations relating to managerial inaction, inconsistent handling of complaints, and perceived protection of senior staff or repeat perpetrators. These comments suggested that concerns about bullying were frequently interpreted not simply as interpersonal conflict issues, but as indicators of broader organisational and leadership failure.

Several respondents described formal reporting processes as lacking transparency, fairness, or independence. Participants frequently said organisational systems seemed more focused on protecting institutional reputation than supporting staff wellbeing or addressing harmful behaviours. Others raised concerns that investigations lacked impartiality or resulted in only limited visible action.

Illustrative comments included:

“Managers need to actually act instead of protecting their friends.” (P088)

“There is no trust in HR because nothing changes.” (P142)

“Policies exist on paper but are not enforced.” (P311)

In addition, participants emphasised the need for stronger leadership visibility, clearer behavioural expectations, and greater accountability for inappropriate conduct irrespective of seniority or professional status.

### Category 3: building psychologically safe workplace cultures

This category focused on participants’ recommendations for creating healthier and psychologically safer workplace environments within UK radiography practice. Respondents consistently emphasised the importance of visible anti-bullying cultures, supportive leadership behaviours, confidential reporting patterns, staff education, and open (vertical and horizontal) communication. Many participants argued that workplace culture improvement required proactive organisational commitment rather than reactive responses after incidents occur.

Several respondents advocated regular training on bullying recognition, respectful communication, bystander organisational initiatives, and speaking-up processes. Others emphasised the importance of anonymous reporting mechanisms and regular opportunities for staff feedback to improve trust in organisational systems. Participants also highlighted the need for organisations to demonstrate visible learning and behavioural change following reported incidents.

Illustrative comments included:

“There should be confidential systems where staff feel safe to report concerns.” (P054)

“Departments need open communication without fear.” (P267)

“Staff should see that concerns actually lead to change.” (P198)

The findings suggest that participants viewed psychologically safe workplace cultures as dependent not only on formal anti-bullying policies, but also on consistent organisational behaviours that promote openness, fairness, support, and accountability.

### Qualitative contextualisation of quantitative findings

The qualitative findings strongly complemented and contextualised the quantitative analyses. Quantitative results showed significant associations between exposure to workplace bullying, diminished Speak-Up Climate, and perceived compromise of patient safety, with mediation analyses suggesting that psychological safety and organisational voice may be key patterns linking bullying to safety-related concerns. The qualitative findings contextualised these patterns by illustrating how fear of retaliation, distrust of organisational reporting systems, perceived leadership inaction, and concerns about anonymity contributed to organisational silence and reduced willingness to raise concerns. Integrating both datasets strengthened interpretation by demonstrating complementary findings across the quantitative and qualitative components. Collectively, the integrated findings suggest that workplace bullying within radiography practice may function not solely as an interpersonal wellbeing issue, but also as a broader organisational culture problem affecting psychological safety, organisational trust, and perceived patient safety.

### Reflexivity

The qualitative free-text analysis was conducted with attention to reflexivity (23, 26). The research team comprised academic, clinical, research, diagnostic and therapeutic radiographers with direct knowledge and experience of UK imaging workplace cultures. This provided contextual sensitivity but also posed a risk of confirmatory interpretation. To mitigate this, the analysts kept reflexive notes during coding to document assumptions, analytic decisions, and potential preconceptions about workplace bullying, reporting culture, and organisational safety. This reflexive stance helped ensure that content categories remained grounded in participants’ written responses rather than being shaped primarily by researchers’ professional expectations.

### Peer debriefing

Peer debriefing strengthened analytical credibility. The wider research team, including members with clinical and academic radiography expertise, reviewed emerging codes and content categories. This process enabled challenging early interpretations, identifying overextended claims, and refining the relationship between qualitative content categories and quantitative findings. Peer debriefing was particularly valuable for ensuring that the qualitative interpretation did not simply duplicate the statistical findings but provided explanatory insight into why fear of retaliation, mistrust of reporting systems, and perceived managerial inaction may undermine speak-up climate.

## Discussion

This study examines the associations among workplace bullying and harassment (WBH), perceived Speak-Up Climate, and perceived compromise of patient safety among radiographers practising in the United Kingdom. Three principal findings emerged. First, greater exposure to workplace bullying was associated with poorer Speak-Up Climate. Second, workplace bullying was associated with greater perceived compromise of patient safety. Third, this association was substantially attenuated after accounting for Speak-Up Climate, with exploratory mediation analyses suggesting a pattern consistent with an indirect association explained by organisational voice and psychological safety. The moderate correlation between the Speak-Up Climate Score and Safety Impact Score supports conceptual relatedness but does not imply construct equivalence; consequently, the observed indirect statistical association should be interpreted as exploratory rather than confirmatory evidence of a distinct organisational process.

The strong association between WBH and a poorer Speak-Up Climate is consistent with psychological safety theory, which posits that interpersonal risk-taking and speaking up become increasingly difficult in threatening workplace environments (1, 27). Exposure to repeated negative acts may foster fear of retaliation, distrust of managerial responsiveness, and expectations of adverse consequences when concerns are raised. These conceptual models are similarly reflected in organisational voice theory, where employees may remain silent when speaking up is perceived as unsafe or futile (2, 28).

The findings also suggest that WBH may extend beyond workforce wellbeing concerns and have implications for patient safety. Previous studies in nursing and medicine have shown that workplace bullying is associated with reduced incident reporting, communication failures, and impaired teamwork (6, 7). The present findings align with this literature and suggest that similar organisational processes may be statistically consistent with radiography practice. Given radiographers’ central role in diagnostic imaging, treatment planning, and multidisciplinary communication, reduced willingness to raise concerns may have important implications for organisational learning and patient safety.

### Workplace bullying and psychological safety

The strongest observed association was between WBH exposure and poorer Speak-Up Climate. Respondents reporting greater exposure to negative acts also reported greater fear of adverse consequences, less confidence in organisational responses and greater reluctance to escalate concerns. These patterns align with psychological safety and organisational silence theories, which propose that perceived interpersonal and organisational risk relates to employee voice and silence. However, the present study measured these variables contemporaneously and cannot determine whether WBH preceded poorer Speak-Up Climate, whether poorer organisational climates increased vulnerability to WBH, or whether the associations were reciprocal (1).

The qualitative findings reinforce this interpretation, highlighting fear of retaliation, reputational damage, and lack of anonymity as central barriers to raising concerns. These patterns align with organisational silence frameworks, which suggest that perceived interpersonal and organisational risk strongly shapes employee voice (2, 28). In this context, bullying appears not only as an interpersonal stressor but also as an organisational factor of workplace communication behaviour.

Notably, the strength of the association between WBH and the speak-up climate (*ρ* ≈ 0.51) suggests that bullying may substantially be associated with organisational voice within radiography. This is consistent with prior healthcare research linking bullying with diminished reporting, disengagement from safety behaviour, and erosion of team communication (6, 7).

### Patient safety implications and indirect association

Although WBH was significantly associated with perceived patient safety compromise, its associations were comparatively modest compared with those associated with speak-up climate. Importantly, after adjusting for speak-up climate, the relationship between WBH and safety impact was no longer statistically significant, while speak-up climate remained strongly associated.

The observed statistical pattern was consistent with an indirect association involving Speak-Up Climate; however, the cross-sectional design does not establish temporal ordering or causal mediation.

These findings are consistent with broader evidence linking psychological safety to safety outcomes. Psychologically unsafe environments are associated with reduced incident reporting, impaired escalation of concerns, and increased likelihood of clinical error (29, 30). Systematic reviews have also demonstrated associations between staff wellbeing, communication breakdown, and patient safety outcomes (8).

From a theoretical perspective, these findings support integrating psychological safety theory (1, 27), Organisational silence models (2, 28), and Psychosocial safety climate theory (1, 27). Together, these frameworks provide a conceptual basis for interpreting the observed associations among WBH, organisational communication, psychological safety and safety-related behaviours.

### Organisational culture, trust, and leadership

The findings highlight the central role of organisational culture and leadership in shaping both exposure to bullying and the speak-up climate. Survey responses indicate low reporting rates and substantial barriers to escalation, including lack of trust, perceived ineffectiveness of reporting systems, and fear of retaliation.

Qualitative data provide further insight, suggesting that staff frequently perceive reporting processes as lacking transparency, independence, and meaningful outcomes. These perceptions matter, as trust in organisational procedures is a key determinant of whether employees feel psychologically safe raising concerns.

This aligns with research demonstrating that procedural justice, leadership credibility, and organisational fairness are strongly associated with employee voice and psychological safety (30). Psychosocial safety climate theory further emphasises that visible organisational commitment to psychological health, including enforcement of policies and accountability for behaviour, is essential for fostering safe communication environments (13).

The present findings therefore suggest that WBH may be associated with both symptoms and correlates of broader organisational culture deficiencies, including weak governance, inconsistent leadership behaviours, and low trust.

### Speak-up climate as a potential organisational priority for prospective evaluation

The observed associations identify Speak-Up Climate as a potentially relevant organisational factor for prospective investigation. Greater WBH exposure was associated with poorer Speak-Up Climate, while poorer Speak-Up Climate was associated with greater perceived Safety Impact after covariate adjustment. These cross-sectional findings do not establish whether improving Speak-Up Climate would reduce WBH or patient-safety concerns; prospective studies evaluating organisational initiatives are required.

The persistence of barriers such as fear of retaliation and lack of confidence in reporting systems reflects wider patterns documented in NHS workforce surveys and public inquiries, where cultures of silence have been implicated in patient harm (12). The present study provides empirical support for these concerns within radiography and suggests that improving psychological safety may be associated with downstream benefits for both staff wellbeing and patient safety.

### Strengths and limitations

This study has several strengths. It draws on a UK-wide volunteer sample of UK-practising radiographers and uses validated instruments, including the NAQ-R, with strong internal consistency. The integration of quantitative and qualitative data enhances interpretive depth and supports qualitative contextualisation of quantitative findings. Additionally, multivariable modelling, sensitivity analyses, and exploratory mediation strengthen the robustness of the results.

However, several limitations remain. Given the cross-sectional design, temporal sequencing and causality cannot be established. The mediation analyses should therefore be interpreted as exploratory evidence of indirect statistical associations rather than a causal conceptual model. Future longitudinal and multi-source studies should combine staff reports with independently collected data on incidents, near-misses, protocol deviations, or safety culture to examine the temporal relationships among workplace bullying, organisational climate, and perceived patient safety outcomes (31). Self-reported measures introduce potential for common-method bias and perceptual inflation.

Additionally, the Safety Impact Score, which measured participants’ perceptions of patient safety compromise, was subjective and did not assess clinical outcomes, adverse events, or incident-reporting data. Consequently, the findings should be interpreted as reflecting a perceived safety climate and organisational safety concerns rather than as evidence of demonstrated patient harm.

The non-probability sampling approach may limit generalisability and overrepresent individuals with experience of bullying. The sample was also skewed towards Black British, Caribbean or African radiographers, and internationally educated radiographers. This may reflect recruitment channels and differential willingness to participate based on prior experience of WBH, limiting generalisability. Direct quantitative comparison with UK radiography data was limited by the absence of a complete, publicly available workforce dataset containing directly equivalent ethnicity, training-location and current-practice variables.

Finally, the Speak-Up Climate Score demonstrated modest internal consistency (Cronbach's *α* = 0.67) and should therefore be interpreted as an exploratory organisational measure requiring further psychometric validation in future studies. The Speak-Up Climate composite demonstrated borderline internal consistency and comparatively modest common-factor coherence. In particular, the reverse-coded organisational-response item had a weak PAF loading and low communality. Although retained because organisational responsiveness was theoretically relevant, this heterogeneity and associated measurement error may have attenuated or otherwise affected the estimated indirect association. Independent validation and confirmatory factor analysis are required before treating the score as an established measure.

### Implications for practice and policy

Psychologically safe reporting environments and anti-retaliation cultures are plausible organisational priorities for prospective evaluation. The findings suggest that organisational initiatives to strengthen the Speak-Up Climate, psychological safety, and supportive leadership are plausible priorities for future workforce development. However, whether these proposed strategies reduce workplace bullying or improve patient safety requires evaluation through prospective and organisational initiative studies. The findings have important implications for healthcare organisations, imaging leaders, and policymakers.

First, organisational initiative to address workplace bullying should extend beyond individual-level strategies to encompass organisational culture, leadership accountability, and system-level change. Evidence suggests that bullying is more likely to be associated with environments characterised by poor leadership, high workload, and weak organisational oversight (7).

Second, strengthening the Speak-Up Climate may be an important organisational strategy worthy of further evaluation, as a poorer Speak-Up Climate was consistently associated with greater perceived compromise of patient safety in this study. Organisations could strengthen confidential, independent reporting mechanisms, protections against retaliation, and transparent organisational responses to concerns. These strategies should be evaluated prospectively.

Third, leadership behaviours are central. Training in psychological safety, respectful workplace practices, and procedural fairness should be embedded within radiography leadership development.

Finally, these findings align with national initiatives such as Freedom to Speak Up, reinforcing the need to embed psychologically safe cultures across NHS services.

## Conclusion

Workplace bullying and harassment among UK radiographers were associated with poorer Speak-Up Climate and greater perceived Safety Impact. After including Speak-Up Climate in the adjusted safety model, the direct association between WBH exposure and perceived Safety Impact was substantially attenuated and no longer statistically significant, while poorer Speak-Up Climate remained associated with greater perceived Safety Impact. The exploratory analysis identified an indirect statistical association involving Speak-Up Climate; however, the cross-sectional design precludes conclusions about temporal ordering or causal mediation. Qualitative findings provided complementary contextual insight, particularly regarding fear of retaliation, distrust of reporting systems and perceived leadership inaction. These findings identify psychologically safe reporting environments, transparent organisational processes and accountability as plausible priorities for prospective evaluation in radiography practice.

## Data Availability

The original contributions presented in the study are included in the article/Supplementary Material, further inquiries can be directed to the corresponding authors.
