# Supplementary material for: Workplace bullying, speak-up climate and perceived patient safety compromise among radiographers practising in the United Kingdom
Source: Front Health Serv. 2026 Oct 19;6:1924715. doi: 10.3389/frhs.2026.1924715 (PMC13724388; doi:10.3389/frhs.2026.1924715)
Supplement: Supplementary file 1 [file Supplementaryfile1.docx]

**Appendix 1: Towards Inclusive Radiography**

**Participant Information Sheet**

**Study title**

Towards Inclusive Radiography: A Quantitative Sequel to a Transnational Scoping Review on Bullying

and Harassment Experiences Among Radiographers

**Background**

Workplace bullying and harassment (WB&H) are psychosocial hazards that affect staff well-being, workforce stability, and patient safety. Radiographers are especially vulnerable due to their frontline roles, lone working, and exposure to stressful clinical environments. A previous scoping review systematically mapped global evidence on the prevalence, types, perpetrators, and impacts of WB&H in radiography. However, the review also identified a knowledge gap: the lack of UK-specific data on radiographers, broken down by perpetrators, types, victims’ characteristics, and coping mechanisms. These data remain limited and fragmented, making targeted actions difficult.

**Aim of your study**

The study aims to create the first national dataset of UK radiographers who have experienced workplace bullying and harassment.

**Who can participate**

The survey is open to all NHS and independent provider radiographers currently practicing in the UK who are either diagnostic or therapeutic professionals. Radiographers who are not HCPC-registered or not practicing, as well as students, are excluded from the survey.

**Is participation voluntary?**

Participation is entirely voluntary, and non-participation is entirely your choice.

**Expectations from participants, if they consent**

If you decide to participate in the study, you will complete a 10-15-minute online questionnaire. There are no right or wrong answers; please answer each question as honestly and accurately as possible, based on your personal experiences, context, perpetrators, and the reporting of workplace bullying and harassment.

**Potential benefits of participation**

This study aims to establish a foundation of evidence to guide national efforts to prevent WB&H and improve psychological safety within imaging departments. It also intends to strengthen workforce retention strategies, in line with the NHS's commitments like Freedom to Speak Up and the People Promise. Furthermore, it will support targeted leadership development programmes inspired by real- world leadership and safety climate concepts, all aimed at creating a safer and more supportive work environment.

**Potential risks of participation**

Any predictable risks arising from this proposed research are considered minimal. If you experience any psychological issues from participating in this research, mental health support services are available through NHS staff services - Staff mental health and wellbeing hubs, NHS England » Staff mental health and wellbeing hubs plus access to Network of support services (NOSS) counselling for NHS staff via your trust occupational health team.

For those who are not NHS employees, there are supportive websites available:

https://www.mind.org.uk/information-support/types-of-mental-health-problems/mental-health-problems-introduction/support-services/

https://www.mentalhealth.org.uk/explore-mental-health/get-help

No physical risks are foreseeable by participating in this study.

**Confidentiality arrangements**

The Data Controller for this study will be Health Sciences University (HSU). The HSU Data Protection Officer provides oversight of HSU activities involving the processing of personal data. All research undertaken at HSU is underpinned by policies and procedures that ensure compliance with the regulations and legislation governing research. This includes data protection legislation: the General Data Protection Regulation (GDPR) and the Data Protection Act (DPA) 2018, which covers how your personal data must be handled.

Your personal data will not be shared with anyone other than the named researchers. Any data that is published will not be able to identify you. To manage the personal data collected from all the studies the University runs, a select number of people outside the research team will have access to your personal data. The Data Protection Officer will need access to your personal data to support you in applying your data subject rights if you wish to.

Special category data, such as race, sexuality, religious beliefs, or physical or mental health status, are not necessary for this study. All the information collected about you during the research will be kept strictly confidential. You will not be able to be identified in any ensuing reports or publications. Confidentiality assurances will be strictly adhered to, subject to legal constraints and professional guidelines. Confidentiality will only be breached if you disclose unsafe practice or a safeguarding concern. In this case, the researcher will report you to the appropriate regulatory body.

All digital copies of research data will be stored in the researcher's password-protected HSU OneDrive account.

Please note that while your responses will be treated confidentially, disclosures of criminal or harmful activity may require reporting to appropriate authorities in line with legal and ethical obligations.

**Data protection**

All data will be stored on the researcher's HSU OneDrive account for ten years. After this period, all the data will be deleted.

**Participants’ rights**

You may withdraw from the study at any stage while completing the questionnaire. Once your answers have been submitted, they are immediately anonymised and cannot be linked back to you. For that reason, withdrawal after submission is not possible.

**Who the funders are**

This study has not received any funding support.

**Ethics Approval**

Ethics approval was obtained from the Health Sciences University Ethics Committee (HRS-2025-

YHAIR).

**Details of who to contact if you have a complaint**

Health Sciences University Ethics Committee

Health Sciences University,

Parkwood Road,

Bournemouth,

BH5 2DF

**Researchers' Contact details**

Chidi Ollawa

Email: chidi.ollawai1@nhs.net

Olanrewaju Lawal

Email: olawal@hsu.ac.uk

**Providing informed consent**

You will have to provide consent via the online survey platform before you are granted access to the questionnaire.

Please ensure you click 'submit' at the end of the survey. Every participant is expected to complete and submit ONLY one survey. Thank you.

**1. Do you agree to participate in this survey?**

*

☐ Yes

☐ No

**2. Are you currently an HCPC-registered radiographer practising in the United Kingdom?**

*

☐ Yes

☐ No

**Demographics**

Let's get to know you.

**3. Age (years):**

☐ Under 25

☐ 25-34

☐ 35-44

☐ 45-54

☐ 55-64

☐ 65+

**4. Gender**:

☐ Male

☐ Female

☐ Non-binary

☐ Prefer not to say

☐ Other (please specify)

5. Ethnicity:

*

☐ Asian / Asian British (Indian » Pakistani » Bangladeshi e Chinese « Any other Asian background)

☐ Black, Black British, Caribbean or African (African * Caribbean « Any other Black, Black British or

Caribbean background)

☐ Mixed / Multiple ethnic groups (White and Black Caribbean e White and Black African « White

and Asian e Any other Mixed / Multiple ethnic background)

☐ White (English, Welsh, Scottish, Northern Irish or British « Irish « Gypsy or Irish Traveller « Roma)

☐ Other ethnic group (Arab or other ethnic group)

6. Country of primary radiography education

*

☐ UK

☐ Non-UK

**7. What is your primary role?**

☐ X-ray/Projectional radiography

☐ CT

☐ MRI

☐ Mammography

☐ Nuclear Medicine

☐ Therapeutic Radiographer

☐ Sonographer

☐ Managers

☐ Practice Educator

☐ DEXA

☐ Reporting Radiographer

☐ Other (please specify)

**8. Primary employment setting**

☐ NHS Trust

☐ Private Hospital/Clinic

☐ University/Academic

☐ Locum/Agency

☐ Other (please specify)

**9. Total years of experience in radiography:**

☐ Less than 1 year

☐ 1-5 years

☐ 6-10 years

☐ 11-15 years

☐ 16-20 years

☐ Over 20 years

**10. Current job level:**

*

☐ Band 5

☐ Band6

☐ Band7

☐ Band 8 or above

☐ Non-AFC role

☐ Other (please specify)

**11. Country of practice:**

☐ England

☐ Scotland

☐ Wales

☐ Northern Ireland

**12. Employment pattern**

☐ Full-time

☐ Part-time

☐ Bank/Locum

☐ In-sourcing companies

☐ Other (please specify)

**Section B: Negative Acts Questionnaire - Revised (NAQ-R) Items [Einarsen et al., (2009)]**

Please indicate how often you have experienced the following behaviours at your current or most recent place of work within the last 6 months.

13. Use the scale below:

1= Never

2 = Now & then

3 = Monthly

4 = Weekly

5 = Daily

*

B1 Someone withholding information which affects your performance or job

☐ 1

☐ 2

☐ 3

☐ 4

☐ 5

B2 Being humiliated or ridiculed in connection with your work

☐ 1

☐ 2

☐ 3

☐ 4

☐ 5

B3 Being asked to fulfil tasks beyond your contractual obligations

☐ 1

☐ 2

☐ 3

☐ 4

☐ 5

B4 Having key areas of responsibility removed or replaced with trivial tasks

☐ 1

☐ 2

☐ 3

☐ 4

☐ 5

B5 Spreading of gossip and rumours about you

☐ 1

☐ 2

☐ 3

☐ 4

☐ 5

B6 Being ignored or excluded from meetings, discussions or the work environment

☐ 1

☐ 2

☐ 3

☐ 4

☐ 5

B7 Someone belittling your work and efforts

☐ 1

☐ 2

☐ 3

☐ 4

☐ 5

B8 Being shouted at or targeted with spontaneous anger

☐ 1

☐ 2

☐ 3

☐ 4

☐ 5

BQ Intimidating behaviours such as finger-pointing, invasion of personal space

☐ 1

☐ 2

☐ 3

☐ 4

☐ 5

B10 Hints or signals that you should quit your job

☐ 1

☐ 2

☐ 3

☐ 4

☐ 5

B11 Repeated reminders of your errors or mistakes

☐ 1

☐ 2

☐ 3

☐ 4

☐ 5

B12 Pressure not to claim something to which you are entitled (e.g. promotion, training, COVID-19 allowance, leave)

☐ 1

☐ 2

☐ 3

☐ 4

☐ 5

B13 Being the target of excessive teasing or sarcasm

☐ 1

☐ 2

☐ 3

☐ 4

☐ 5

B14 Being exposed to an unmanageable workload

☐ 1

☐ 2

☐ 3

☐ 4

☐ 5

B15 Threats of violence or actual physical abuse

☐ 1

☐ 2

☐ 3

☐ 4

☐ 5

B16 Repeated exaggerated negative reports to your supervisor/Lead by people you don't get along with

☐ 1

☐ 2

☐ 3

☐ 4

☐ 5

B17 Verbal abuse from non-staff eg. patients’ relatives/escorts/students.

☐ 1

☐ 2

☐ 3

☐ 4

☐ 5

B18 Being excluded from learning/training / CPD/Conference opportunities

☐ 1

☐ 2

☐ 3

☐ 4

☐ 5

B19 Obstruction when escalating safety concerns or incident reporting.

☐ 1

☐ 2

☐ 3

☐ 4

☐ 5

B20 Undermining or disparaging comments on professional online/social platforms

☐ 1

☐ 2

☐ 3

☐ 4

☐ 5

B21 Pressure to perform additional imaging/treatments/work contrary to protocol/scope of practice

☐ 1

☐ 2

☐ 3

☐ 4

☐ 5

B22 Repeated deliberate use of incorrect pronouns or disrespect of your preferred form of address

☐ 1

☐ 2

☐ 3

☐ 4

☐ 5

**Section C: Bullying Experience, Perpetrators, Context and Reporting**

Bullying at work usually involves repeated, unwelcome, deliberate or unconscious negative actions directed at a member of staff who is vulnerable (Einarsen & Agotnes, 2023).

Common examples of bullying and harassing behaviour include mobbing, physical intimidation, physical abuse, verbal abuse, gaslighting, passive aggression, isolation, sabotaging work, deliberately withholding information to get someone in trouble or make them look incompetent, playing jokes designed to embarrass or humiliate, and lying/spreading rumours against a target.

14. Have you ever been bullied or harassed in your role as a Radiographer in the UK (in

the last 6 months)?

*

☐ Yes

☐ No

☐ Unsure

☐ Other (please specify)

15. Have you witnessed others being bullied in your workplace (in the last 6 months)?

*

☐ Yes

☐ No

16. Where did the bullying occur?

*

☐ Control/Treatment room

☐ Scan room

☐ Reporting room

☐ Ward/Theatre

☐ Online (e.g. Departmental WhatsApp group, Facebook, Linkedin)

☐ Staff room

☐ Not applicable

☐ Office

☐ Other (please specify)

17. When did the bullying most often occur?

*

☐ Daytime

☐ Evenings

☐ Nights

☐ Weekends

☐ On-call

☐ All the time eg. online and workplace

☐ Not applicable

☐ Other (please specify)

18. Were others present when the behaviours occurred?

*

☐ Yes, Patients

☐ Yes, Patient Relatives

☐ Yes, Staff

☐ Yes, Student

☐ Not applicable

☐ No

☐ Other (please specify)

19. How long have the bullying behaviours persisted (if applicable)?

*

☐ Less than 1 month

☐ 1-6 months

☐ 6-12 months

☐ Over 1 year

☐ Not applicable

☐ Other (please specify)

20. Who was the perpetrator(s)? (Tick all that apply):

*

☐ Line manager/supervisor

☐ Colleague of the same level

☐ Senior colleague

☐ Junior colleague

☐ Patient or member of the public

☐ Admin staff

☐ Not applicable

☐ Other (please specify)

21. Did you formally report the bullying/harassment?

*

☐ Yes, to HR

☐ Yes, to the line manager

☐ Yes, HCPC

☐ Yes, SOR or other trade union

☐ Yes, via local incident reporting channels (e.g., Datix, freedom to speak up guardian, whistleblowing)

☐ No

☐ Not Applicable

22. If you did not report the bullying/harassment or delayed reporting, what were the reason(s)? (Tick all that apply):

*

☐ Fear of retaliation

☐ Lack of trust in the reporting system

☐ Thought nothing would change

☐ Didn't recognise it as bullying at the time

☐ Did not know how to report

☐ Too busy with work and other things

☐ Considered it as “part of the job"

☐ Not Applicable

☐ Yes other (please specify details)

23. If reported, was the matter addressed satisfactorily?

*

☐ Yes

☐ No

☐ Partially

☐ Still ongoing

☐ Don't know/not informed of outcome

☐ Not Applicable

☐ Other (please specify)

**Section D: Impact of Bullying and Organisational Support**

24. To what extent has bullying impacted your mental health?

*

☐ Not at all

☐ Slightly

☐ Moderately

☐ Severely

25. Has bullying led you to consider leaving your job?

*

☐ Yes

☐ No

☐ Already left

26. Does your organisation have a clear anti-bullying policy?

*

☐ Yes

☐ No

☐ Not sure

27. Do you feel safe and supported at your workplace?

*

☐ Always

☐ Often

☐ Sometimes

☐ Rarely

☐ Never

28. Have you received training or guidance on recognising and dealing with bullying and harassment?

*

☐ Yes

☐ No

☐ Not sure

**SECTION E — Psychological & Occupational Outcomes [GHQ-12; Goldberg & Williams, (1988)].**

Please indicate how often you have experienced the following at your current or most recent place of work.

Note: these questions are related to bullying and harassment at work ONLY.

29. Use the scale below:

1 = More than usual

2 = Same as usual

3 = Less than usual

*

E1, Unable to concentrate?

☐1

☐ 2

☐ 3

E2. Disrupted sleep due to worry?

☐1

☐ 2

☐ 3

E3. Feeling like a useless member of your team?

☐1

☐ 2

☐ 3

E4. Incapable of making decisions?

☐1

☐ 2

☐ 3

E5. Felt constantly under strain?

☐1

☐ 2

☐ 3

E6. Felt you couldn't overcome your difficulties?

☐1

☐ 2

☐ 3

E7. Unable to enjoy your normal day-to-day activities?

☐1

☐ 2

☐ 3

E8. Unable to face up to problems?

☐1

☐ 2

☐ 3

EQ. Feeling unhappy or depressed?

☐1

☐ 2

☐ 3

E10. Losing confidence in yourself?

☐1

☐ 2

☐ 3

E11. Thinking of yourself as a worthless person?

☐1

☐ 2

☐ 3

E12. Feeling unhappy in your role?

☐1

☐ 2

☐ 3

30. Absence/Presenteeism (working whilst unwell)

E13. In the LAST 12 MONTHS, how many WORKDAYS did you miss due to work-related stress, bullying, or harassment? [Number of days - if none, use '0']

31. E14. In the LAST 12 MONTHS, how many days did you work while unwell (presenteeism) due to workload/pressure? [Number of days - if none, use '0']

*

**SECTION F — Patient Safety & Professional Impact [Sexton et al. (2006)].**

Please indicate which of these best expresses your perception of the impact of bullying and harassment behaviours at your current workplace.

32. Please use the scale below to rate the following statements:

1 = Disagree strongly

2 = Disagree slightly

3 = Neutral

4 = Agree slightly

5 = Agree strongly

These statements relate to how bullying and harassment behaviours affect patient care and staff performance.

*

F1. Due to bullying and harassment it is difficult to discuss errors

☐1

☐ 2

☐ 3

☐ 4

☐ 5

F2. Bullying and harassment discourages teamwork and cooperation among staff

☐1

☐ 2

☐ 3

☐ 4

☐ 5

F3. | am more likely to make errors in tense and hostile work situations caused by bullying and harassmdent

☐1

☐ 2

☐ 3

☐ 4

☐ 5

F4. Stress from bullying and harassment adversely affects my performance at work

☐1

☐ 2

☐ 3

☐ 4

☐ 5

F5. Communication breakdowns caused by bullying and harassment can lead to delays in service delivery and

negatively affect patient care

☐1

☐ 2

☐ 3

☐ 4

☐ 5

F6. Bullying and harassment reduces staff willingness to support one another when caring for patients

☐1

☐ 2

☐ 3

☐ 4

☐ 5

33. Please use the scale below to rate the following statements:

1 = Strongly disagree

2 = Disagree

3 = Neither agree nor disagree

4 = Agree

5 = Strongly agree

These statements relate to how safe you feel raising concerns, including concerns about bullying, harassment, or patient safety.

*

F7. lam less likely to raise safety concerns when | fear negative reactions from colleagues or managers.

☐1

☐ 2

☐ 3

☐ 4

☐ 5

F8. In my department, it is difficult to speak up about concerns with patient care

☐1

☐ 2

☐ 3

☐ 4

☐ 5

F9. | may hesitate to report bullying, harassment, or safety incidents because i am concerned about being

identified.

☐1

☐ 2

☐ 3

☐ 4

☐ 5

F10. I feel confident that concerns raised in my department are addressed fairly and promptly.

☐1

☐ 2

☐ 3

☐ 4

☐ 5

34. Please use the scale below to rate the following statements:

1 = Strongly disagree

2 = Disagree

3 = Neither agree nor disagree

4 = Agree

5 = Strongly agree

The following statement relates to perceptions of professional regulatory processes.

*

F11. In some cases, workplace bullies exploit the Health and Care Professions Council (HCPC) referral process to make malicious or vexatious complaints against radiographers.

☐1

☐ 2

☐ 3

☐ 4

☐ 5

**Section G: Open-Ended Comments (Optional)**

35. Please feel free to suggest ways your organisation could better prevent bullying or support staff facing bullying: ………………………………………………………

36. The next stage of this study is the qualitative phase, which will include more detailed discussions about your responses and experiences with WBH.

If you are interested in participating in the upcoming (qualitative) phase, please leave your email address below, and we may get in touch with you. Please note that by providing your email, you are only giving us permission to contact you; you are not automatically consenting to participate in the next phase. Thank you.

**Debriefing sheet for participants**

**Investigators**: Mr Chidi Ollawa and Dr Olanrewaju Lawal

Thank you again for taking the time to participate in this study.

**What was the purpose of the study?**

The study aims to generate the first professional-specific national dataset on the prevalence, typology, and organisational determinants of workplace bullying and harassment (WB&H) among radiographers in the UK.

**What happens now?**

The study will provide an evidence base for national guidance on preventing WB&H and promoting psychological safety in imaging departments. Workforce-retention strategies aligning with NHS Freedom to Speak Up and People Promise priorities. Targeted leadership development modules informed by authentic leadership and safety climate constructs. Please email the researchers ([chidi.ollawal@nhs.net](mailto:chidi.ollawal@nhs.net), olawal@aecc.ac.uk), if you wish to receive a copy of the article.

**Who should | contact for further information?**

Please contact the researchers, Mr Chidi Ollawa- chidi.ollawal1@nhs.net, and Dr Olanrewaju Lawal — OLawal@aecc.ac.uk, if you have any questions, complaints or concerns.

If you do feel any psychological issues from taking part in this research, there are mental health support services available for NHS staff services - Staff mental health and wellbeing hubs, NHS England » Staff mental health and wellbeing hubs plus access to Network of support services (NOSS) counselling for NHS staff via your trust occupational health team.

For those that are not NHS employees, there are supportive websites available:

[https://www.mind.org.uk/information-support/types-of-mental-health-problems/mental-health- problems-introduction/support-services/](https://www.mind.org.uk/information-support/types-of-mental-health-problems/mental-health-%20problems-introduction/support-services/) <https://www.mentalhealth.org.uk/explore-mental-health/get-help>

Dr Olanrewaju Lawal

Health Sciences University

Parkwood Campus

Parkwood Road

Bournemouth BH5 2DF

Email: [olawal@hsu.ac.uk](mailto:olawal@hsu.ac.uk)

Any ethical concerns about the study may be directed to the School of Research Ethics lead, Dr Hazreen Majid (hazreen.majid@hsu.ac.uk)

**Thank you again for your participation in this study and for supporting research at Health Sciences University.**
