# Supplementary material for: Workplace bullying, speak-up climate and perceived patient safety compromise among radiographers practising in the United Kingdom
Source: Front Health Serv. 2026 Oct 19;6:1924715. doi: 10.3389/frhs.2026.1924715 (PMC13724388; doi:10.3389/frhs.2026.1924715)
Supplement: Supplementary file 2 [file Table1.docx]

**SUPPLEMENTARY TABLES**

**Table S1:** Relationship between companion manuscripts Phases 2 and 3

| **Characteristic** | **Companion manuscript (Reference 4)** | **Present manuscript** |
| --- | --- | --- |
| Primary aim | Psychological and occupational consequences of workplace bullying and harassment | Organisational safety implications of workplace bullying and harassment |
| Research Questions | *1. Is exposure to workplace bullying and harassment associated with greater psychological distress among radiographers?*  *2. Is exposure to workplace bullying and harassment associated with higher rates of sickness absence and presenteeism?*  *3. Do these associations vary by ethnicity and country of professional training?* | *1. Is exposure to workplace bullying associated with a perceived poorer speak-up climate?*  *2. Is exposure to workplace bullying linked to a higher perception of patient safety compromise?*  *3. To what extent is the association between workplace bullying and perceived patient safety compromise statistically accounted for by Speak-Up Climate?* |
| Primary outcomes | GHQ-12 psychological distress validated scale, presenteeism, absenteeism, intention to leave | Safety Impact Score, Speak-Up Climate, reporting behaviour, organisational prevention strategies |
| Main regression outcome | Psychological distress / occupational outcomes | Safety Impact and Speak-Up Climate |
| Mediation analysis | No | Yes (Speak-Up Climate as mediator) |
| Qualitative analysis | None | Free-text responses on organisational prevention and support |
| Main contribution | Individual consequences | Organisational safety and safety culture |
| Principal findings | A total of 385 radiographers participated. Behaviourally defined WBH exposure (based on NAQ-R criteria) was reported by 41.8%, and 30.9% met criteria for probable psychological distress. Weekly or daily WBH exposure was independently associated with probable psychological distress (adjusted odds ratio [aOR] 7.41, 95% CI 4.38–12.54, p < 0.001), any sickness absence (aOR 3.67, 95% CI 2.05–6.55, p < 0.001), and any presenteeism (aOR 4.38, 95% CI 2.67–7.18, p < 0.001). Dose–response analyses showed progressively poorer psychological and occupational outcomes as NAQ-R scores increased. |  |

**Table S2**: Preliminary psychometric evaluation of study-specific composite measures

| **Scale** | **Item** | **Corrected item-total correlation** |
| --- | --- | --- |
| Safety Impact | Q32_1 | 0.544 |
|  | Q32_2 | 0.724 |
|  | Q32_3 | 0.769 |
|  | Q32_4 | 0.753 |
|  | Q32_5 | 0.790 |
|  | Q32_6 | 0.802 |
| Speak-Up Climate | Q33_1 | 0.436 |
|  | Q33_2 | 0.508 |
|  | Q33_3 | 0.626 |
|  | Q33_4 (reverse coded) | 0.258 |

**Table S3**: Exploratory Indirect Association Result

| **Effect** | **B** | **95% CI** | **p** |
| --- | --- | --- | --- |
| Total association, c | 0.316 | 0.179–0.452 | <0.001 |
| a association: NAQ-R → Speak-Up Climate | 0.698 | 0.555–0.841 | <0.001 |
| b association: Speak-Up Climate → Safety Impact, adjusted for NAQ-R | 0.378 | 0.255–0.501 | <0.001 |
| Direct association, c′ | 0.052 | −0.095–0.199 | 0.488 |
| Indirect statistical association, a × b | 0.264 | bootstrap CI 0.169–0.373 | — |

**Table S4:** Missing data and composite score calculation

| **Measure** | **Item-level missing (%)** | **Eligible for composite** | **Person-mean prorated** | **Excluded (below threshold)** |
| --- | --- | --- | --- | --- |
| Safety Impact Score (6 items) | 0.0–3.6 | 372 (96.6%) | 6 (1.6%) | 13 (3.4%) |
| Speak-Up Climate Score (4 items) | 0.0–0.3 | 384 (99.7%) | 1 (0.3%) | 1 (0.3%) |

**Table S5:** Sensitivity analyses for adjusted regression models

| **Sample** | **Model** | **n** | **Key predictor** | **B** | **95% CI** | **p** |
| --- | --- | --- | --- | --- | --- | --- |
| **Full sample** | NAQ-R → Speak-up | 380 | NAQ-R | 0.693 | 0.556–0.829 | <0.001 |
| **Full sample** | NAQ-R → Safety | 368 | NAQ-R | 0.308 | 0.173–0.443 | <0.001 |
| **Full sample** | NAQ-R + Speak-up → Safety | 367 | NAQ-R | 0.048 | -0.096–0.192 | 0.513 |
| **Full sample** | NAQ-R + Speak-up → Safety | 367 | Speak-up | 0.370 | 0.250–0.490 | <0.001 |
| **NHS-only** | NAQ-R + Speak-up → Safety | 308 | NAQ-R | 0.057 | -0.106–0.221 | 0.490 |
| **NHS-only** | NAQ-R + Speak-up → Safety | 308 | Speak-up | 0.376 | 0.247–0.505 | <0.001 |
| **UK-trained** | NAQ-R + Speak-up → Safety | 163 | NAQ-R | 0.134 | -0.120–0.388 | 0.301 |
| **UK-trained** | NAQ-R + Speak-up → Safety | 163 | Speak-up | 0.428 | 0.260–0.596 | <0.001 |
| **Diagnostic-only** | NAQ-R + Speak-up → Safety | 310 | NAQ-R | -0.002 | -0.162–0.158 | 0.984 |
| **Diagnostic-only** | NAQ-R + Speak-up → Safety | 310 | Speak-up | 0.391 | 0.250–0.532 | <0.001 |

**Table S6:** Bootstrapped indirect effects across sensitivity samples

| **Sample** | **n** | **Indirect effect** | **95% bootstrap CI** |
| --- | --- | --- | --- |
| Full sample | 367 | 0.259 | 0.166–0.365 |
| NHS-only | 308 | 0.262 | 0.160–0.389 |
| UK-trained | 163 | 0.287 | 0.166–0.438 |
| Diagnostic-only | 310 | 0.270 | 0.163–0.394 |

g

**Table S7:** Comparison of primary and complete-case adjusted regression models

| **Model** | **Analysis** | **N** | **R²** | **Adjusted R²** |
| --- | --- | --- | --- | --- |
| Model 1: Speak-Up Climate outcome | Primary person-mean-prorated analysis | 369 | 0.250 | 0.231 |
| Model 1: Speak-Up Climate outcome | Complete-case analysis | 353 | 0.259 | 0.240 |
| Model 2: Safety Impact outcome | Primary person-mean-prorated analysis | 358 | 0.118 | 0.096 |
| Model 2: Safety Impact outcome | Complete-case analysis | 337 | 0.120 | 0.096 |
| Model 3: Safety Impact outcome including Speak-Up Climate | Primary person-mean-prorated analysis | 357 | 0.230 | 0.208 |
| Model 3: Safety Impact outcome including Speak-Up Climate | Complete-case analysis | 337 | 0.223 | 0.199 |

**Table S8:** Comparison of focal coefficients in primary and complete-case adjusted models

| **Model and predictor** | **Analysis** | **N** | **B** | **HC3 SE** | **95% CI** | **p** |
| --- | --- | --- | --- | --- | --- | --- |
| Model 1: NAQ-R mean | Primary | 369 | 0.688 | 0.070 | 0.550–0.825 | <0.001 |
| Model 1: NAQ-R mean | Complete case | 353 | 0.686 | 0.071 | 0.546–0.825 | <0.001 |
| Model 2: NAQ-R mean | Primary | 358 | 0.316 | 0.070 | 0.179–0.453 | <0.001 |
| Model 2: NAQ-R mean | Complete case | 337 | 0.305 | 0.070 | 0.167–0.442 | <0.001 |
| Model 3: NAQ-R mean | Primary | 357 | 0.052 | 0.075 | −0.095–0.199 | 0.488 |
| Model 3: NAQ-R mean | Complete case | 337 | 0.054 | 0.078 | −0.098–0.207 | 0.487 |
| Model 3: Speak-Up Climate | Primary | 357 | 0.378 | 0.063 | 0.255–0.501 | <0.001 |
| Model 3: Speak-Up Climate | Complete case | 337 | 0.366 | 0.065 | 0.238–0.494 | <0.001 |

**Note**: ‘B’ denotes an unstandardised regression coefficient. NAQ-R was analysed as a mean score ranging from 1 to 5. Higher Speak-Up Climate scores represent a poorer perceived Speak-Up Climate.

**Table S9.** Exploratory indirect statistical association in the primary and complete-case samples

| **Analysis** | **N** | **Indirect association** | **95% bootstrap CI** |
| --- | --- | --- | --- |
| Primary person-mean-prorated analysis | 357 | 0.264 | 0.169–0.374 |
| Complete-case analysis | 337 | 0.250 | 0.154–0.364 |

**Note**: Confidence intervals were estimated using 5,000 percentile bootstrap resamples. The analysis is exploratory and cross-sectional and should not be interpreted as establishing temporal or causal mediation.
